# Coumarin linked to 2-phenylbenzimidazole derivatives as potent α-glucosidase inhibitors

**DOI:** 10.1038/s41598-024-57673-z

**Published:** 2024-03-28

**Authors:** Mina Sadeghi Ganjeh, Ali Mazlomifar, Ashraf Sadat Shahvelayti, Shiva Khalili Moghaddam

**Affiliations:** 1 Department of Chemistry, College of Basic Sciences, Yadegar-e-Imam Khomeini (RAH) Shahre Rey Branch, Islamic Azad University, Tehran, Iran; 2 Department of Biology, College of Basic Sciences, Yadegar-e-Imam Khomeini (RAH) Shahre Rey Branch, Islamic Azad University, Tehran, Iran

**Keywords:** 2-Phenylbenzimidazole, Coumarin, α-Glucosidase, Molecular modeling, Biochemistry, Computational biology and bioinformatics, Drug discovery

## Abstract

α-Glucosidase inhibitors have emerged as crucial agents in the management of type 2 diabetes mellitus. In the present study, a new series of coumarin-linked 2-phenylbenzimidazole derivatives **5a–m** was designed, synthesized, and evaluated as anti-α-glucosidase agents. Among these derivatives, compound **5k** (IC_50_ = 10.8 µM) exhibited a significant inhibitory activity in comparison to the positive control acarbose (IC_50_ = 750.0 µM). Through kinetic analysis, it was revealed that compound **5k** exhibited a competitive inhibition pattern against α-glucosidase. To gain insights into the interactions between the title compounds and α-glucosidase molecular docking was employed. The obtained results highlighted crucial interactions that contribute to the inhibitory activities of the compounds against α-glucosidase. These derivatives show immense potential as promising starting points for developing novel α-glucosidase inhibitors.

## Introduction

Diabetes mellitus is a chronic metabolic disorder characterized by persistent hyperglycemia resulting from defects in insulin secretion, insulin action, or both. It is a significant global health concern affecting millions of individuals worldwide and is associated with various complications, including cardiovascular disease, nephropathy, neuropathy, and retinopathy^1,2^. Diabetes mellitus encompasses different types, such as type 1 diabetes, type 2 diabetes, gestational diabetes, and other specific forms. Type 1 diabetes is an autoimmune disease that destroys pancreatic β-cells, leading to an absolute insulin deficiency. In contrast, type 2 diabetes, the most prevalent form, arises from a combination of insulin resistance and impaired insulin secretion. Gestational diabetes occurs during pregnancy and typically resolves after childbirth^3^. The pathophysiology of type 2 diabetes involves the development of insulin resistance, wherein target cells exhibit reduced responsiveness to insulin. To compensate for this resistance, the pancreas increases insulin secretion, resulting in elevated insulin levels in the blood. Over time, excessive insulin production can lead to β-cell exhaustion and a decline in insulin production^4,5^.

α-Glucosidase (EC 3.2.1.20) is a hydrolase enzyme found in the intestinal cells at the brush border surface^6^. α-Glucosidase plays a crucial role in the small intestine's final steps of carbohydrate digestion. It breaks down the α-1,4 glycosidic bonds present in complex carbohydrates, such as disaccharides and polysaccharides, releasing absorbable monosaccharides, including glucose. This enzymatic activity regulates postprandial blood glucose levels. Inhibition of α-glucosidase effectively slows down the digestion of complex carbohydrates, leading to reduced glucose absorption and a blunted glycemic response after meals^7^. α-Glucosidase inhibitors like acarbose and miglitol are widely used as oral anti-diabetic medications to manage type 2 diabetes. However, the currently approved α-glucosidase inhibitors have notable side effects such as abdominal distention, bloating, and diarrhea^8^. Consequently, scientists have been actively pursuing discovering and developing new compounds that can offer enhanced therapeutic benefits while minimizing adverse effects. They aim to identify and synthesize novel inhibitors that improve treatment outcomes and reduce the risk of undesirable reactions^6,9^.

**Figure 1:**
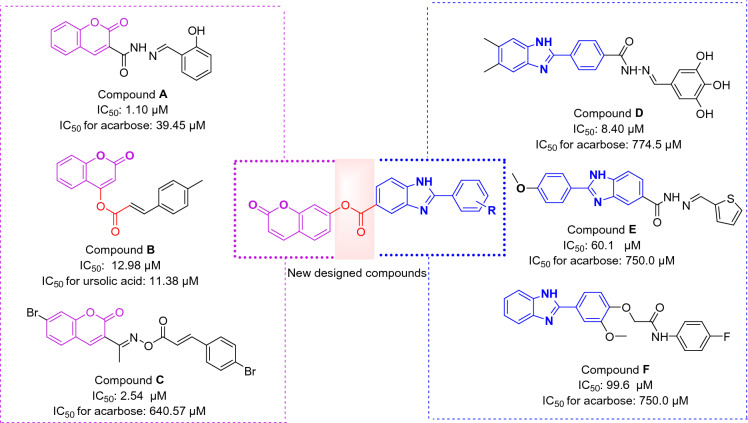
Rationale for the design of coumarin linked to 2-phenylbenzimidazole derivatives as the new α-glucosidase inhibitors.

Coumarin, a naturally occurring compound, and its derivatives have demonstrated various pharmacological activities, including antioxidant, anti-inflammatory, antimicrobial, and anticancer properties^10–14^. These characteristics make it an attractive pharmacophore for designing and developing new compounds with potential therapeutic applications^15^. In the case of α-glucosidase inhibition, coumarin derivatives **A–C** (Fig. 1) demonstrated α-glucosidase inhibitory activities^16–18^. As can be seen in Fig. 1, coumarincarbohydrazone derivative **A** with hydroxy substituent on 2-position of pendant phenyl group was 35.8-fold more potent than standard inhibitor acarbose. Moreover, in α-glucosidase inhibitor **B** coumarin by a carboxylate linker attached to styrene (Fig. 1). It is worthy to note that coumarin derivative **C** was 252-fold more potent than acarbose (Fig. 1). Furthermore, the potency of anti- α-glucosidase activity of coumarin derivatives was also confirmed in other studies^19–21^.

Benzoimidazole is a nitrogen containing heterocycle that has gained significant attention in medicinal chemistry due to its diverse biological activities and therapeutic potential^22^. It exhibits various pharmacological properties, including antimicrobial, anticancer, anti-inflammatory, and antioxidant activities^23–26^. Also, the benzoimidazole is a valuable structural motif in design of α-glucosidase inhibitors^27–29^. For example, 2-phenylbenzimidazole derivative **D** exhibited excellent inhibitory activity yeast α-glucosidase (IC_50_ = 8.40 μM comparing with acarbose IC_50_ = 774.5 μM)^30^. Furthermore, 2-phenylbenzimidazole derivatives **E** and **F** showed high anti-α-glucosidase activities in comparison to standard inhibitor^31,32^.

In view of the mentioned scaffolds, herein, by connection of coumarin to 2-phenylbenzimidazole by carboxylate linker, a novel series of α-glucosidase inhibitors was designed. Next, all designed compounds were examined against yeast α-glucosidase. Finally, in silico assessments were done to get insight into the structure–activity relationships (SARs) and binding interaction modes of the title compounds.

## Results and discussion

### Chemistry

**Scheme 1:**
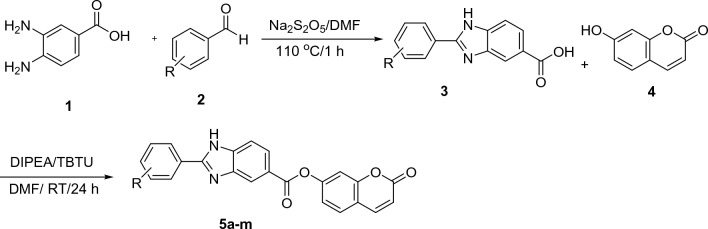
Synthesis of coumarin linked to 2-phenylbenzimidazole derivatives **5a–m**.

Synthesis of the target compounds **5a–m** was schematically described in Scheme 1. Briefly, 3,4-aminobenzoic acid **1** was added to different aldehydes **2** in DMF, and the mixture was stirred at 110 °C for 1 h to synthesize 2-phenylbenzimidazole-5-carboxylic acids **3**. Next, intermediate **3** was reacted with 7-hydroxy-2H-chromen-2-one **4** in the presence of *N*,*N*-diisopropylethylamine (DIPEA), and 2-(1*H*-Benzotriazole-1-yl)-1,1,3,3-tetramethyluronium tetrafluoroborate (TBTU) in DMF to synthesize final products **5a–m**. FTIR, ^1^H-NMR, and ^13^C-NMR characterized all synthesized compounds.

### In vitro α-glucosidase inhibition and SAR discussion

The potency of the target compounds **5a–m** was evaluated in vitro against α-glucosidase, compared with acarbose used as a positive control. The IC_50_ values obtained for each compound are listed in Table 1. Notably, compound **5k**, which contains a 2-hydroxyphenyl moiety, exhibited promising activity with an IC_50_ value of 10.8 ± 0.1 µM, making it the most potent inhibitor compared to acarbose.

**Table 1:** α-Glucosidase inhibition assay of the target compounds **5a–m**.


Compound	R	IC_50_ (µM)^a^	Compound	R	IC_50_ (µM)^a^
**5a**	H	50.0 ± 0.8	**5h**	2,3-diCl	45.2 ± 0.2
**5b**	4-F	30.6 ± 0.4	**5i**	4-CH_3_	44.9 ± 0.0
**5c**	4-Cl	26.9 ± 1.1	**5j**	4-OCH_3_	40.3 ± 0.5
**5d**	4-Br	22.9 ± 0.2	**5k**	2-OH	10.8 ± 0.1
**5e**	2-Cl	34.7 ± 0.7	**5l**	2-CH_3_-3-NO_2_	119.5 ± 0.1
**5f**	3-Br	25.2 ± 0.5	**5m**	2-CH_3_-3-Cl	40.9 ± 1.1
**5g**	2,4-diF	46.7 ± 0.2	**Acarbose**^b^		750.0 ± 2.0

^a^Data represented in terms of mean ± SD.

^b^Positive control.

In the present study, compound **5a**, an un-substituted derivative (R=H), demonstrated an IC_50_ value of 50.0 ± 0.8 µM, which was approximately 15-fold better than acarbose.

Different moieties were substituted at the R position to explore SAR and optimization of the inhibitory activity. Initially, halogens were introduced at the *para* position of the phenyl ring, and the following potency order was observed: 4-Br (**5d**, IC_50_ = 22.9 ± 0.2 µM) > 4-Cl (**5c**, IC_50_ = 26.9 ± 1.1 µM) > 4-F (**5b**, IC_50_ = 30.6 ± 0.4 µM).

Substituting chlorine at the *ortho* position (**5e**; IC_50_ = 34.7 ± 0.7 µM) or bromine at the *meta* position (**5f.**; IC_50_ = 34.7 ± 0.7 µM) resulted in reduced activity compared to their *para*-substituted counterparts. However, despite the decrease in activity, these derivatives still exhibited better inhibition compared to un-substituted derivative **5a**.

Furthermore, it was observed that the introduction of second fluorine atom on 4-fluorophenyl ring of compound **5b** and the second chlorine atom on 2-chlorophenyl ring of derivative **5e**, as in compounds **5g** and **5h**, respectively, reduced anti-α-glucosidase activity.

SAR study also demonstrated that electron-withdrawing groups at the *para* position of the phenyl ring are more effective than electron-donating groups (halogen derivatives **5b–d** vs. methyl and methoxy derivatives **5i–j**). In contrast, 2-hydroxy derivative **5k** was significantly more active than 2-chlorophenyl derivative **5e**.

Moreover, SAR study of compounds with two deference substitutions, involving both electron-donating and electron-withdrawing groups, was performed. The lowest potency was observed in 2-CH_3_-3-NO_2_ derivative **5l**, with an IC_50_ value of 119.5 ± 0.1 µM. Replacing of 3-NO_2_ group of compound **5l** with 3-Cl substituent, as in compound **5m**, improved the potency.

**Figure 2:**
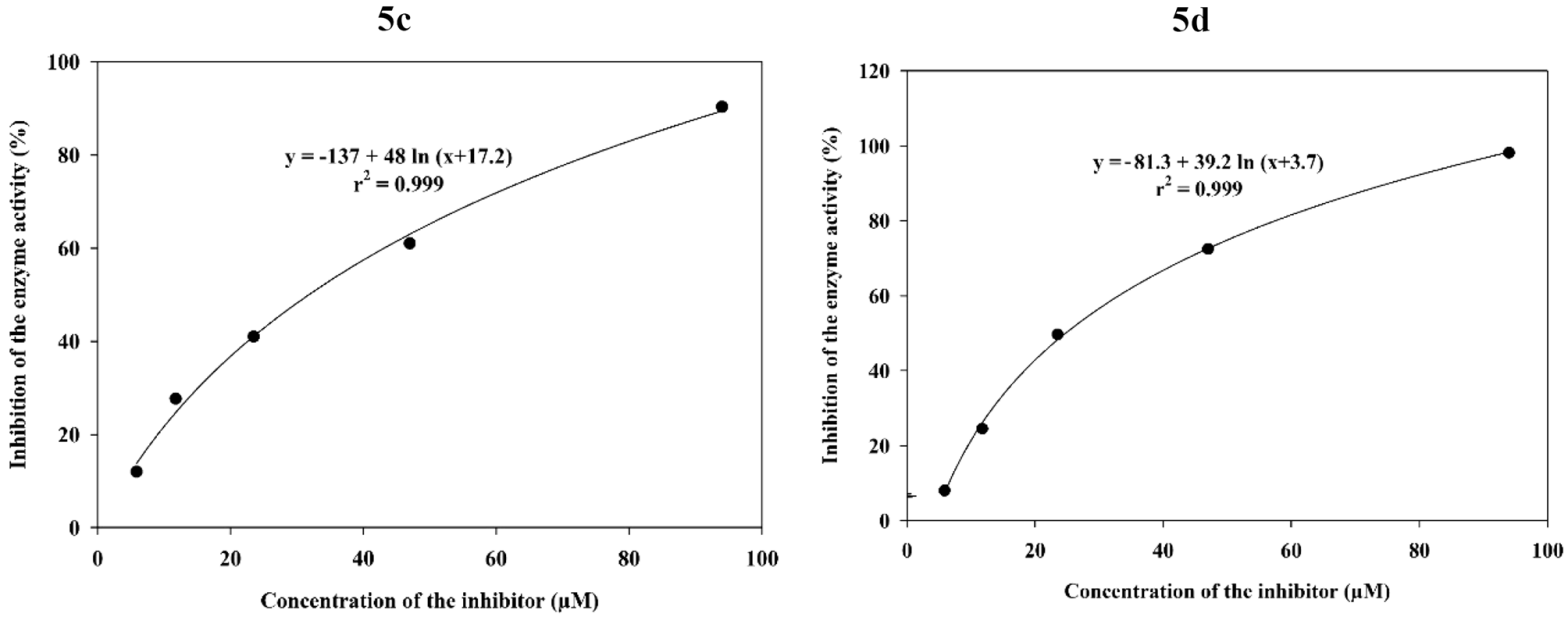
Dose response curves of compounds **5c** and **5d**.

In addition to the listed of IC_50_ values in Table 1, for example, two dose response curves for two compounds **5c** and **5d** were showed in Fig. 2.

### Enzyme kinetic studies

**Figure 3:**
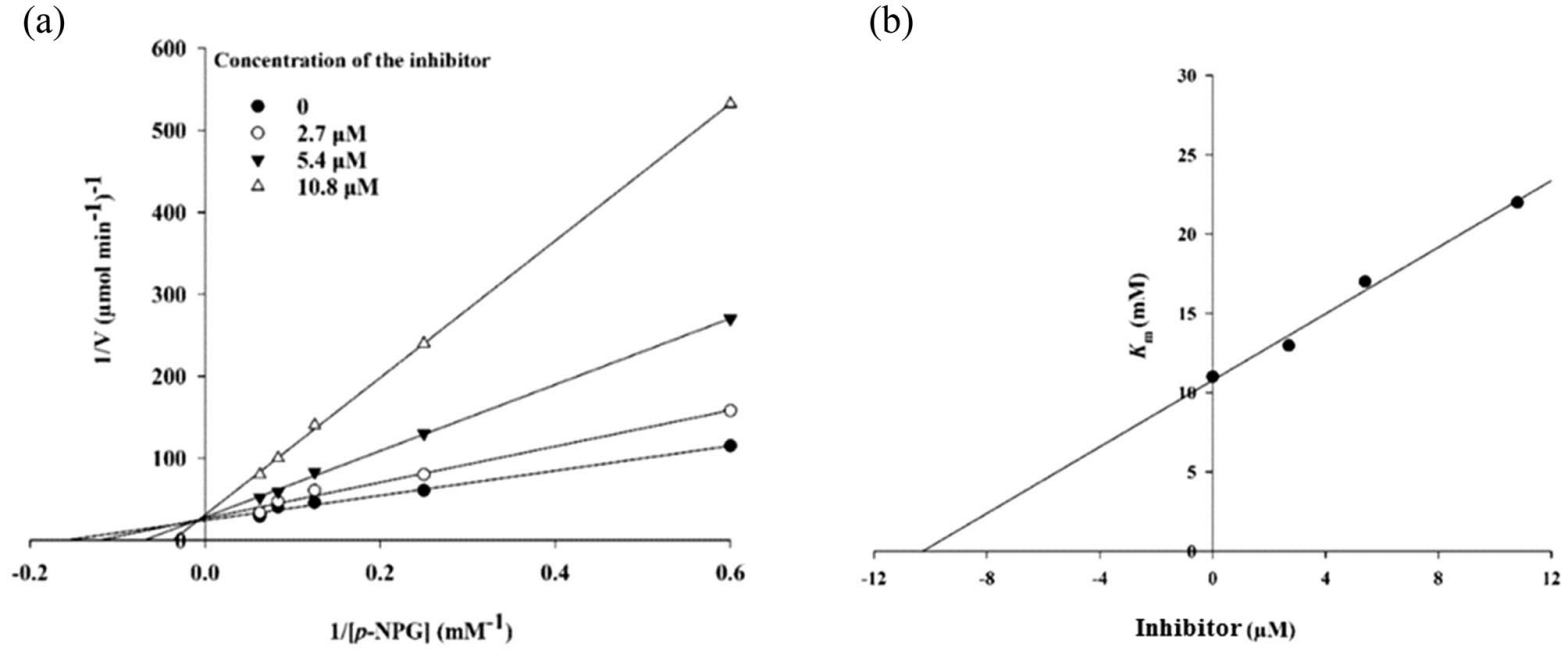
Kinetics of α-glucosidase inhibition by **5k** (inhibitor): (**a**) the Lineweaver–Burk plot in the absence and presence of different concentrations of **5k**; (**b**) the secondary plot between *K*_m_ and various concentrations of **5k**.

According to Fig. 3a, the Lineweaver–Burk plot showed that the Michaelis–Menten constant (*K*_m_) gradually increased and maximum velocity (V_max_) remained unchanged with increasing inhibitor concentration indicating a competitive inhibition. The results show **5k** binds to the active site on the enzyme and compete with the substrate for binding to the active site. Furthermore, the plot of the *K*_m_ versus different concentrations of inhibitor gave an estimate of the inhibition constant (K_i_) of 10.4 µM (Fig. 3b).

### Molecular docking

**Figure 4:**
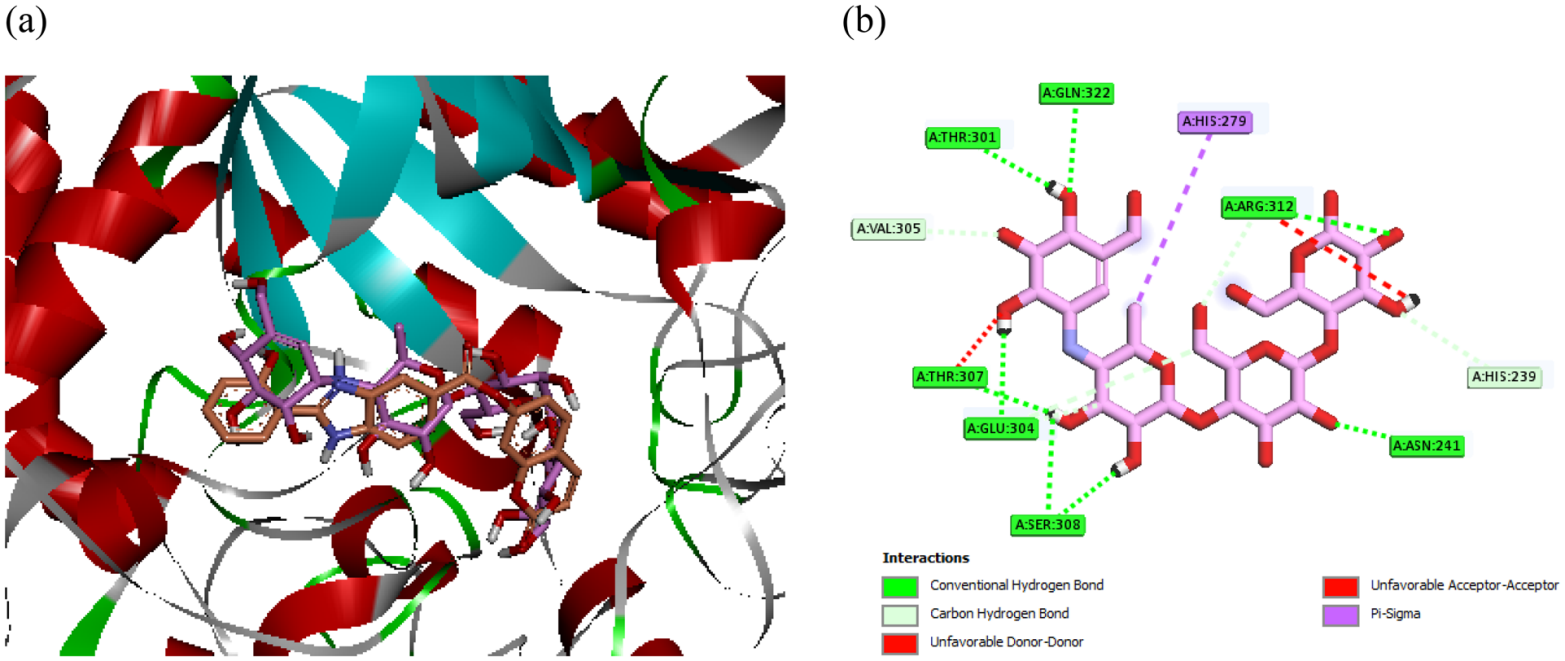
(**a**) Acarbose (pink) and compound **5k** (orange) in the α-glucosidase active site and (**b**) interaction mode of acarbose.

In the enzyme assay section, it was reported that the assay was conducted utilizing the enzyme *Saccharomyces cerevisiae* α-glucosidase (EC. 3. 2. 1. 20). However, due to the unavailability of the 3D crystallographic structure of this enzyme in the corresponding databases, a new structure was developed using homology modeling^33^. After that, based on obtained mode of representative compound (the most potent compound **5k**) in the kinetic study (competitive mode), docking study of the target compounds was performed in the α-glucosidase active site. Superimposed structure of the standard inhibitor (acarbose, pink) and the most potent compound **5k** (orange) is showed in Fig. 4a. as can be seen in Fig. 4b, acarbose as positive control established hydrogen bonds with residues Thr307, Thr301, Asn241, Glu304, Ser308, Phe157, and Pro309, Arg312, and Gln322, non-classical hydrogen bonds with residues Val305, His239, Arg312, and Glu304, a hydrophobic interaction with His279, and two unfavorable interactions with residues Thr307 and Arg312 in the α‐glucosidase active site.

**Figure 5:**
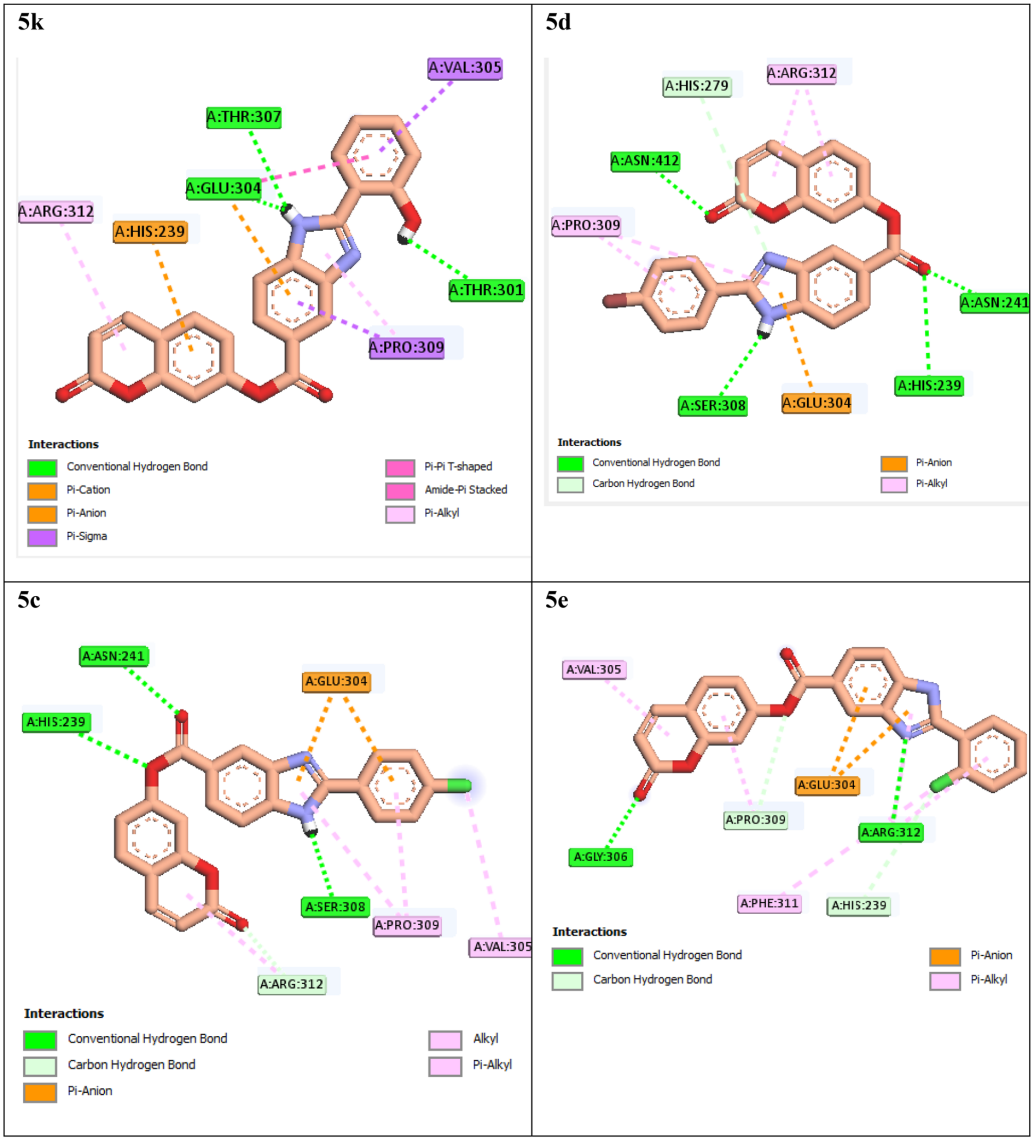
Interaction modes of compounds **5c–e** and **5k** in the α-glucosidase active site.

**Figure 6:**
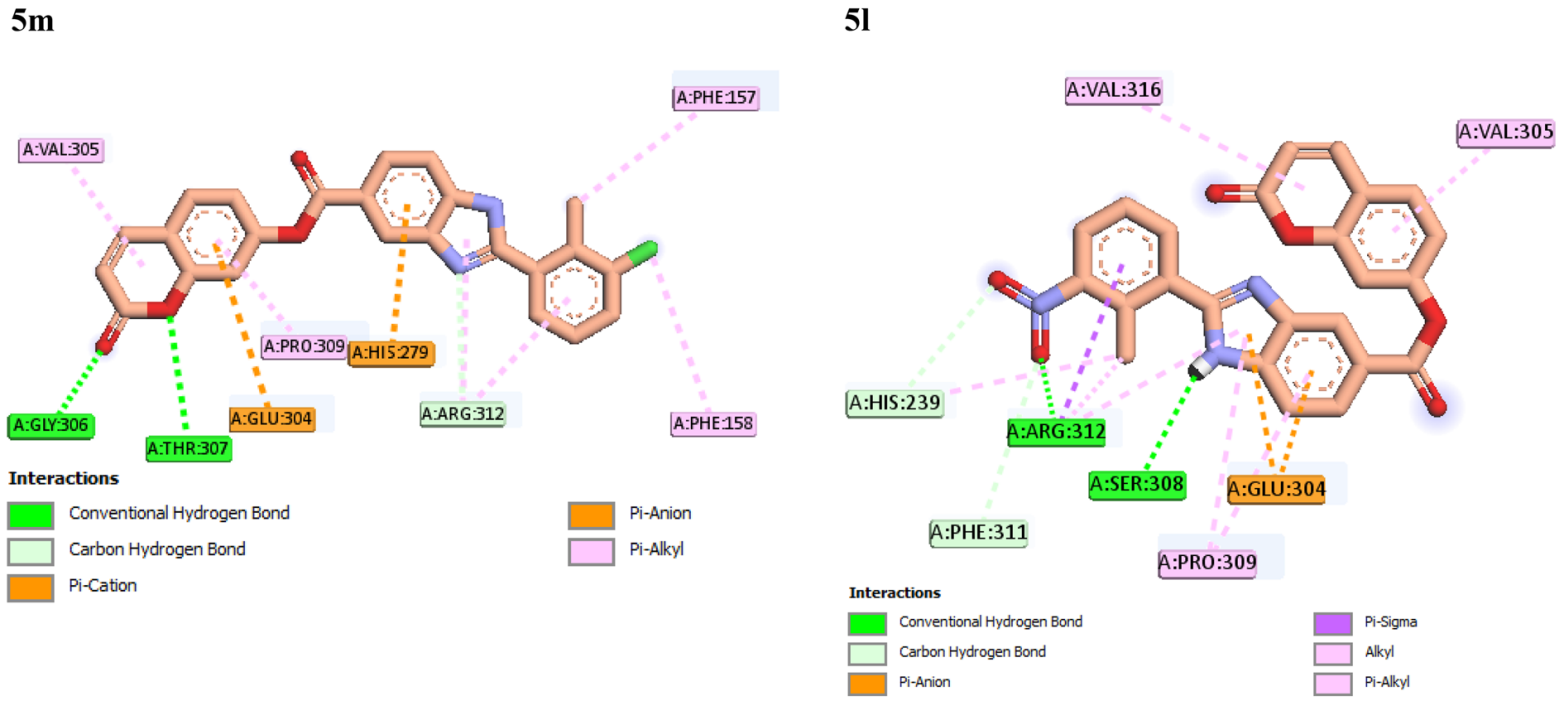
Interaction modes of compounds **5m** and **5l** in the α-glucosidase active site.

For this study, we considered the compounds **5c–e**, **5k**, and **5l–m** because they were either potent or had interesting points in term of SARs. Interaction modes of compounds **5c–e**, **5k**, and **5l–m** were showed in Figs. 5 and 6 and binding energies of these compounds and acarbose were listed in Table 2.

**Table 2:** Binding energies of the selected compounds **5c–e**, **5k**, and **5l-m**.

Compound	Binding energy (Kcal/mol)
**5k**	−10.8
**5d**	−9.49
**5c**	−9.41
**5e**	−9.34
**5m**	−9.13
**5l**	−8.72

The comparison of binding energies of the compounds **5c–e**, **5k**, and **5l–m** with acarbose revealed that these new compounds can be bind to the active site easier than the standard inhibitor (Table 2). These results were confirmed by the obtained results of in vitro assessment.

Interaction mode of the most potent compound **5k** showed that this compound established three hydrogen bonds with residues Thr307, Thr301 (via 2-hydroxy group), and Glu304 (Fig. 5). This compound also formed a π-anion interaction with Glu304 and a π-cation interaction with His239. Moreover, hydrophobic interactions between compound **5k** and residues Glu304, His239, Val305, Pro309, and Arg312 were observed. In vitro study demonstrated that compound **5k** with 2-hydroxy substituent was twofold more potent thah the second potent compound **5d** with 4-bromo substituent (Table 1). The comparison of binding energies of these compounds showed that compound **5k** had lower binding energy in comparison to compound **5d**. Compound **5d** formed four classical hydrogen bonds with residues Asn241, Asn412, Ser308, and His239 and a non-classical hydrogen bond with His279 (Fig. 5). Glu304 established a π-anion interaction with compound **5d**. Furthermore, the latter compound created hydrophobic interactions with residues Arg312 and Pro309. Replacement of 4-bromo substituent of compound **5d** with 4-chloro substituent, in case of compound **5c**, led to a negligible decrease in inhibitory activity and a negligible increase in binding energy (Table 1 and 2). As can be seen in Fig. 5, 4-chloro derivative **5c** formed three classical hydrogen bonds (with Asn241, His239, and Ser308) and a non-classical hydrogen bond (with Arg312) with the active site of α-glucosidase. This compound established π-anion interactions with Glu304 and hydrophobic interactions with Arg312, Pro309, and Val305. The comparison of IC_50_ values of 4-chloro derivative **5c** and 2-chloro derivatives **5e** demonstrated that translocation of chlorine atom on the 2-phenyl ring had a moderate effect on anti-α-glucosidase activity, and 4-chloro was preferred (Table 1). The obtained binding energies of compounds **5c** and **5e** were in agreement with in vitro study (Table 2). The comparison of binding modes of the latter compounds showed that 4-chloro derivative established three classical hydrogen bonds and a non-classical hydrogen bond while 2-chloro derivative formed two classical hydrogen bonds with Gly306 and Arg312 and two non-classical hydrogen bonds with His239 and Pro309. According to the mode of interaction of acarbose (Fig. 4b), it seems that Gly306 is not very important in the occurrence of the effect. π-Anion interactions were similar in both of compounds **5c** and **5e**. Moreover, hydrophobic interactions also were similar in these compound only compound **5e** interacted with Phe311 while compound **5c** interacted Arg312.

As can be seen Table 1, replacement of 3-chloro substituent of compound **5m** with 3-nitro substituent, as in case of compound **5l**, the inhibitory activity diminished to three folds. This finding with obtained binding energies for these compounds was confirmed. Binding mods of compound **5m** and **5l** showed that compound **5m** formed interactions with nine amino acids Val305, Gly306, Thr307, Glu304, Pro309, His279, Arg312, Phe158, and Phe157 and compound **5l** established interactions with eight amino acids His239, Arg312, Phe311, Ser308, Pro309, Glu304, Val305, and Val316 (Fig. 6).

## Conclusion

Following our expertise in the rational design of α-glucosidase inhibitors, a series of coumarin-benzimidazole derivatives were designed and synthesized. The chemical structures of these derivatives were thoroughly characterized using various analytical techniques, including ^1^H-NMR, ^13^C-NMR, and FTIR analysis. Our results revealed that all the synthesized compounds exhibited significant anti-α-glucosidase activity, as demonstrated by their IC_50_ values ranging from 10.8 ± 0.1 μM to 119.5 ± 0.1 μM, in comparison to the reference compound acarbose (IC_50_ = 750.0 µM). These findings highlight the efficacy of the designed backbone in targeting α-glucosidase. Among the tested compounds, compound **5k** emerged as the most potent inhibitor with an IC_50_ value of 10.8 ± 0.1 μM. Further kinetic experiments revealed that compound **5k** exhibited a competitive inhibition pattern. To gain deeper insights into the interaction mechanism of these derivatives at the α-glucosidase active site, we performed molecular docking study. By this study, the observed SARs were rationalized and possible binding modes were detected.

## Experimental

### General synthesis of 2-aryl-1*H*-benzo[*d*]imidazole-5-carboxylic acids 3

A mixture of 3,4-aminobenzoic acid **1** (1 mmol) and aldehydes **2** (1 mmol) in DMF (10 mL) was stirred at 110 °C for 1 h. Then, water was added to the reaction mixture and observed participates were separated by filtration to give pure 2-aryl-1*H*-benzo[*d*]imidazole-5-carboxylic acids **3**.

### General synthesis of the target compounds 5a–m

A mixture of 2-aryl-1*H*-benzo[*d*]imidazole-5-carboxylic acids **3** (1 mmol), 7-hydroxy-2H-chromen-2-one **4** (1 mmol), DIPEA (1.1 mmol), and TBTU (1 mmol) in DMF (15 mL) was stirred at room temperature for 24 h. Then, cold water was added to the reaction mixture and formed participates were separated by filtration to give pure target compounds **5a–m**.

### 2-Oxo-2H-chromen-7-yl 2-phenyl-1H-benzo[d]imidazole-5-carboxylate (5a)

Brown solid; yield: 65%; MP = 180–183 °C; IR (KBr, v_max_) 3320(NH), 3040 (C–H aromatic), 1680(C = O) cm^−1^;^1^H NMR (400 MHz, DMSO-*d*_6_) δ 10.51 (s, 1H, NH_Imidazole_), 8.34 (s, 1H, H_Ar_), 8.00 (d, *J* = 9.5 Hz, 1H, H_Ar_), 7.79 (d, *J* = 7.6 Hz, 1H, H_Ar_), 7.65 (d, *J* = 8.6 Hz, 1H, H_Ar_), 7.59 (d, *J* = 8.0 Hz, 2H, H_Ar_), 7.45 (d, *J* = 7.6 Hz, 1H, H_Ar_), 7.34 (t, *J* = 7.8 Hz, 2H, H_Ar_), 7.20 (s, 1H, H_Ar_), 7.09 (t, *J* = 7.4 Hz, 1H, H_Ar_), 7.05 (dd, *J* = 8.6, 2.4 Hz, 1H, H_Ar_), 6.31 (d, *J* = 9.5 Hz, 1H, H_Ar_). ^13^C NMR (101 MHz, DMSO-*d*_*6*_) δ 164.64, 161.62, 160.76, 155.79, 152.95, 147.45, 144.79, 142.25, 138.87, 136.74, 130.00, 129.41, 127.19, 124.26, 123.43, 119.66, 118.79, 115.08, 113.40, 113.14, 113.05, 102.01.

### 2-Oxo-2H-chromen-7-yl 2-(4-fluorophenyl)-1H-benzo[d]imidazole-5-carboxylate (5b)

Cream solid; yield:81%; MP = 195–197 °C; IR (KBr, v_max_) 3401 (NH), 3065(CH aromatic), 1670 (C=O) cm^−1^;^1^H NMR (400 MHz, DMSO-*d*_6_) δ 10.57 (s, 1H, NH_Imidazole_), 8.33 (s, 1H, H_Ar_), 8.01 (d, *J* = 9.5 Hz, 1H, H_Ar_), 7.80 (d, , *J* = 7.9 Hz, 1H, H_Ar_), 7.66 (d, *J* = 8.6 Hz, 1H, H_Ar_), 7.63–7.57 (m, 2H, H_Ar_), 7.46 (d, , *J* = 8.1 Hz, 1H, H_Ar_), 7.22–7.15 (m, 3H, H_Ar_), 7.05 (dd, *J* = 8.7, 2.4 Hz, 1H, H_Ar_), 6.32 (d, *J* = 9.5 Hz, 1H, H_Ar_). ^13^C NMR (101 MHz, DMSO-*d*_6_) δ 164.60, 161.62, 160.76, 159.90, 157.51, 155.80, 152.91, 147.17, 144.80, 142.22, 138.74, 135.27, 135.25, 130.01, 127.15, 125.19, 123.24, 121.52, 121.44, 119.16, 117.70, 116.13, 115.91, 113.41, 113.15, 113.05, 102.01.

### 2-Oxo-2H-chromen-7-yl 2-(4-chlorophenyl)-1H-benzo[d]imidazole-5-carboxylate (5c)

Cream solid; yield:68%; MP = 180–182 °C; IR (KBr, v_max_) 3235 (NH), 3030 (CH Aromatic), 1684 (C=O) cm^−1^;^1^H NMR (400 MHz, DMSO-*d*_6_) δ 8.88 (s, 1H, NH_Imidazole_), 8.27 (s, 1H, H_Ar_), 8.01 (d, *J* = 9.5 Hz, 1H, H_Ar_), 7.86 (d, *J* = 7.2 Hz, 1H, H_Ar_), 7.65 (d, *J* = 8.6 Hz, 1H, H_Ar_), 7.52 (d, *J* = 7.2 Hz, 1H, H_Ar_), 7.37–7.29 (m, 2H, H_Ar_), 7.24–7.13 (m, 3H, H_Ar_), 7.04 (dd, *J* = 8.7, 2.4 Hz, 1H, H_Ar_), 6.32 (d, *J* = 9.4 Hz, 1H, H_Ar_). ^13^C NMR (101 MHz, DMSO-*d*_6_) δ 165.92, 162.95, 161.62, 160.76, 160.54, 155.79, 152.13, 144.80, 142.12, 139.68, 135.42 (d, *J* = 3.0 Hz), 130.00, 129.92, 129.84, 127.04, 125.90, 123.05, 119.14, 115.68, 115.47, 113.40, 113.14, 113.04, 101.99.

### 2-Oxo-2H-chromen-7-yl 2-(4-bromophenyl)-1H-benzo[d]imidazole-5-carboxylate (5d)

Brown solid; yield: 65%; MP = 179–181 °C; IR (KBr, v_max_) 3336(NH), 3030 (C–H Aromatic), 1651(C=O) cm^−1^; ^1^H NMR (400 MHz, DMSO-*d*_6_) δ 10.65 (s, 1H, NH_Imidazole_), 8.33 (s, 1H, H_Ar_), 8.01 (d, *J* = 9.5 Hz, 1H, H_Ar_), 7.84 (d, *J* = 8.1 Hz, 1H, H_Ar_), 7.66 (d, *J* = 8.7 Hz, 1H, H_Ar_), 7.61–7.50 (m, 4H, H_Ar_), 7.36 (d, *J* = 7.9 Hz, 1H, H_Ar_), 7.20 (s, 1H, H_Ar_), 7.05 (dd, *J* = 8.6, 2.4 Hz, 1H, H_Ar_), 6.32 (d, *J* = 9.5 Hz, 1H, H_Ar_). ^13^C NMR (101 MHz, DMSO-d6) δ 164.88, 161.62, 160.76, 155.79, 152.93, 147.62, 144.80, 142.23, 138.24, 132.25, 130.01, 127.16, 125.07, 123.08, 121.60, 115.89, 113.41, 113.15, 113.05, 102.01.

### 2-Oxo-2H-chromen-7-yl 2-(2-chlorophenyl)-1H-benzo[d]imidazole-5-carboxylate (5e)

Cream solid; yield: 68%; MP = 189–191 °C; IR (KBr, v_max_) 3221 (NH), 3025 (CH aromatic), 1679 (C=O) cm^−1^;^1^H NMR (400 MHz, DMSO-*d*_6_) δ 10.13 (s, 1H, NH _Imidazole_), 8.34 (s, 1H, H_Ar_), 7.99 (d, *J* = 9.5 Hz, 1H, H_Ar_), 7.84 (d,* J* = 7.5 Hz, 1H, H_Ar_), 7.75 (d, *J* = 7.5 Hz, 1H, H_Ar_), 7.64 (d, *J* = 8.6 Hz, 1H, H_Ar_), 7.53 (d, *J* = 7.5 Hz, 1H, H_Ar_), 7.43 (d,* J* = 7.5 Hz, 1H, H_Ar_), 7.34 (t, *J* = 7.4 Hz, 1H, H_Ar_), 7.23 (d, *J* = 7.4 Hz, 1H, H_Ar_), 7.19 (s, 1H, H_Ar_), 7.04 (dd, *J* = 8.6, 2.4 Hz, 1H, H_Ar_), 6.31 (d, *J* = 9.5 Hz, 1H, H_Ar_). ^13^C NMR (101 MHz, DMSO-d6) δ 165.36, 161.60, 160.76, 155.79, 152.86, 146.56, 144.76, 142.26, 138.61, 134.61, 125.32, 123.25, 119.57, 115.34, 113.39, 113.13, 113.04, 101.99.

### 2-Oxo-2H-chromen-7-yl 2-(3-bromophenyl)-1H-benzo[d]imidazole-5-carboxylate (5f)

Cream solid; yield: 66%; MP = 172–174 °C; IR (KBr, v_max_) 3243 (NH), 3050 (CH aromatic), 1679 (C=O) cm^−1^; ^1^H NMR (400 MHz, DMSO-*d*_6_) δ 10.70 (s, 1H, NH_Imidazole_), 8.33 (s, 1H, H_Ar_), 8.01 (d, *J* = 9.5 Hz, 1H, H_Ar_), 7.93 (s, 1H, H_Ar_), 7.80 (d,* J* = 7.8 Hz, 1H, H_Ar_), 7.66 (d, *J* = 8.7 Hz, 1H, H_Ar_), 7.54 (d, *J* = 7.5 Hz, 1H, H_Ar_), 7.49 (d, *J* = 7.3 Hz, 2H, H_Ar_), 7.37–7.26 (m, 2H, H_Ar_), 7.20 (s, 1H, H_Ar_), 7.05 (dd, *J* = 8.7, 2.4 Hz, 1H, H_Ar_), 6.32 (d, *J* = 9.5 Hz, 1H, H_Ar_). ^13^C NMR (101 MHz, DMSO-d6) δ 165.11, 161.62, 160.76, 155.80, 152.74, 148.19, 144.79, 142.24, 140.41, 137.08, 131.45, 130.00, 127.16, 126.91, 125.48, 123.81, 122.13, 122.05, 118.48, 115.58, 113.40, 113.15, 113.05, 102.00.

### 2-Oxo-2H-chromen-7-yl 2-(2,4-difluorophenyl)-1H-benzo[d]imidazole-5-carboxylate (5g)

Brown solid; yield: 65%; MP = 201–203 °C; IR (KBr, v_max_) 3343(NH), 3045 (C–H aromatic), 1654 (C=O) cm^−1^; ^1^H NMR (400 MHz, DMSO-*d*_6_) δ 10.37 (s, 1H, NH_Imidazole_), 8.33 (s, 1H, H_Ar_), 7.99 (d, *J* = 9.5 Hz, 1H, H_Ar_), 7.92–7.82 (m, 1H, H_Ar_), 7.78 (d,* J* = 7.8 Hz, 1H, H_Ar_), 7.64 (d, *J* = 8.6 Hz, 1H, H_Ar_), 7.49 (d,* J* = 7.9 Hz, 1H, H_Ar_), 7.44–7.31 (m, 1H, H_Ar_), 7.18 (s, 1H, H_Ar_), 7.08 (t, *J* = 8.7 Hz, 1H, H_Ar_), 7.03 (dd, *J* = 8.6, 2.4 Hz, 1H, H_Ar_), 6.31 (d, *J* = 9.5 Hz, 1H, H_Ar_). ^13^C NMR (101 MHz, DMSO-d6) δ 165.32, 161.60, 160.76, 160.40, 160.29, 157.98, 157.86, 155.78, 155.60, 155.47, 153.13, 153.00, 146.81, 144.75, 142.24, 139.57, 129.97, 127.17, 125.77, 125.74, 125.67, 125.64, 122.52, 122.49, 122.40, 122.37, 113.38, 113.13, 113.04, 111.89, 111.85, 111.67, 111.64, 105.03, 104.79, 104.76, 104.53, 101.99.

### 2-Oxo-2H-chromen-7-yl 2-(2,3-dichlorophenyl)-1H-benzo[d]imidazole-5-carboxylate (5h)

Cream solid; yield: 71%; MP = 182–184 °C; IR (KBr, v_max_) 3364 (NH), 3035(CH aromatic) 1661 (C=O) cm^−1^;^1^H NMR (400 MHz, DMSO-*d*_6_) δ 10.28 (s, 1H, NH_Imidazole_), 8.34 (s, 1H, H_Ar_), 7.99 (d, *J* = 9.5 Hz, 1H, H_Ar_), 7.84 (d, *J* = 7.9 Hz, 1H, H_Ar_), 7.73 (d, *J* = 8.0 Hz, 1H, H_Ar_), 7.64 (d, *J* = 8.6 Hz, 1H, H_Ar_), 7.49 (d, *J* = 8.0 Hz, 1H, H_Ar_), 7.37 (t, *J* = 8.1 Hz, 1H, H_Ar_), 7.27 (d, *J* = 8.0 Hz, 1H, H_Ar_), 7.18 (s, 1H, H_Ar_), 7.03 (d, *J* = 8.2 Hz, 1H, H_Ar_), 6.30 (d, *J* = 9.4 Hz, 1H, H_Ar_). ^13^C NMR (101 MHz, DMSO-d6) δ 165.54, 161.59, 160.75, 155.78, 152.12, 146.53, 144.76, 138.22, 136.59, 132.48, 129.98, 128.61, 127.56, 127.28, 125.44, 124.93, 122.06, 113.39, 113.14, 113.04, 101.99.

### 2-Oxo-2H-chromen-7-yl 2-(*p*-tolyl)-1H-benzo[d]imidazole-5-carboxylate (5i)

Cream solid; yield: 71%; MP = 193–195 °C; IR (KBr, v_max_) 3213 (NH), 3020 (CH aromatic), 2965 (CH aliphatic), 1671 (C=O) cm^−1^;^1^H NMR (400 MHz, DMSO-*d*_6_) δ 10.43 (s, 1H, NH _Imidazole_), 8.33 (s, 1H, H_Ar_), 8.00 (d, *J* = 9.5 Hz, 1H, H_Ar_), 7.83 (d, *J* = 7.3 Hz, 1H, H_Ar_), 7.82 (d, *J* = 7.4 Hz, 1H, H_Ar_), 7.65 (d, *J* = 8.6 Hz, 1H, H_Ar_), 7.47 (d, *J* = 8.4 Hz, 2H, H_Ar_), 7.33 (d,* J* = 7.1 Hz, 1H, H_Ar_), 7.19 (s, 1H, H_Ar_), 7.14 (d, *J* = 8.2 Hz, 2H, H_Ar_), 7.04 (dd, *J* = 8.6, 2.4 Hz, 1H, H_Ar_), 6.31 (d, *J* = 9.5 Hz, 1H, H_Ar_)2.25(s, 3H, CH_3_). ^13^C NMR (101 MHz, DMSO-d6) δ 164.37, 161.62, 160.76, 155.79, 147.43, 144.78, 142.20, 139.37, 136.36, 133.23, 129.99, 129.77, 127.15, 119.67, 116.32, 113.39, 113.14, 113.04, 102.00, 20.92.

### 2-Oxo-2H-chromen-7-yl 2-(4-methoxyphenyl)-1H-benzo[d]imidazole-5-carboxylate (5j)

Cream solid; yield: 68%;MP = 169–171 °C; IR (KBr, v_max_) 3355 (NH), 3070(CH aromatic), 2965(CH aliphatic), 1670 (C=O) cm^−1^; ^1^H NMR (400 MHz, DMSO-*d*_6_) δ 10.37 (s, 1H, NH_Imidazole_), 8.32 (s, 1H, H_Ar_), 8.01 (d, *J* = 9.5 Hz, 1H, H_Ar_), 7.88–7.80 (d, *J* = 7.4 Hz, 1H, H_Ar_), 7.66 (d, *J* = 8.6 Hz, 1H, H_Ar_), 7.55–7.45 (m, 2H, H_Ar_), 7.39–7.30 (d, *J* = 7.3 Hz, 1H, H_Ar_), 7.23–7.18 (m, 1H, H_Ar_), 7.05 (dd, *J* = 8.7, 2.4 Hz, 1H, H_Ar_), 6.91 (d, *J* = 9.0 Hz, 2H, H_Ar_), 6.32 (d, *J* = 9.5 Hz, 1H, H_Ar_), 3.73 (s, 3H, OCH_3_). ^13^C NMR (101 MHz, DMSO-*d*_6_) δ 164.09, 161.63, 160.76, 155.99, 155.80, 153.19, 145.93, 144.80, 142.18, 131.96, 130.01, 127.14, 125.15, 123.60, 121.21, 119.56, 114.49, 113.41, 113.15, 113.05, 102.01, 55.63.

### 2-Oxo-2H-chromen-7-yl 2-(2-hydroxyphenyl)-1H-benzo[d]imidazole-5-carboxylate (5k)

Brown solid;Yield:70%;MP = 187–189 °C; IR (KBr, v_max_) 3320(NH), 3030 (C–H aromatic), 1650 (C=O) cm^−1^;^1^H NMR (400 MHz, DMSO-*d*_6_) δ 11.08 (s, 1H, OH), 8.88 (s, 1H, NH_Imidazole_), 8.28 (s, 1H, H_Ar_), 8.01 (d, *J* = 9.5 Hz, 1H, H_Ar_), 7.85 (d,* J* = 7.8 Hz, 1H, H_Ar_), 7.65 (d, *J* = 8.6 Hz, 1H, H_Ar_), 7.53 (d,* J* = 7.6 Hz, 1H, H_Ar_), 7.38–7.32 (m, 2H, H_Ar_), 7.32–7.24 (m, 2H, H_Ar_), 7.20 (d, *J* = 2.4 Hz, 1H, H_Ar_), 7.04 (dd, *J* = 8.6, 2.4 Hz, 1H, H_Ar_), 6.31 (d, *J* = 9.5 Hz, 1H, H_Ar_). ^13^C NMR (101 MHz, DMSO-*d*_6_) δ 165.88, 161.63, 160.76, 158.15, 155.79, 152.87, 147.25, 144.80, 142.11, 139.17, 130.00, 128.85, 127.87, 127.49, 127.05, 125.06, 123.65, 119.66, 115.78, 113.40, 113.14, 113.04, 101.99.

### 2-Oxo-2H-chromen-7-yl 2-(2-methyl-3-nitrophenyl)-1H-benzo[d]imidazole-5-carboxylate (5l)

Cream solid; yield: 71%;MP = 194–196 °C; IR (KBr, v_max_) 3375 (NH), 3045(CH aromatic), 2960(CH aliphatic), 1665 (C=O) cm^−1^; ^1^H NMR (400 MHz, DMSO-*d*_6_) δ 10.29 (s, 1H, NH_Imidazole_), 8.35 (s, 1H, H_Ar_), 8.00 (d, *J* = 9.5 Hz, 1H, H_Ar_), 7.84 (d,* J* = 7.4 Hz, 1H, H_Ar_), 7.76 (d, *J* = 8.1 Hz, 1H, H_Ar_), 7.71 (d, *J* = 8.0 Hz, 1H, H_Ar_), 7.64 (d, *J* = 8.6 Hz, 1H, H_Ar_), 7.55 (d,* J* = 7.4 Hz, 1H, H_Ar_), 7.45 (t, *J* = 8.1 Hz, 1H, H_Ar_), 7.19 (s, 1H, H_Ar_), 7.04 (dd, *J* = 8.6, 2.4 Hz, 1H, H_Ar_), 6.31 (d, *J* = 9.5 Hz, 1H, H_Ar_), 2.30 (s, 3H, CH_3_). ^13^C NMR (101 MHz, DMSO-*d*_*6*_) δ 165.49, 161.60, 160.75, 155.78, 152.54, 151.36, 144.77, 142.32, 138.76, 137.75, 130.29, 129.99, 127.29, 127.18, 126.99, 121.69, 113.40, 113.14, 113.04, 101.99, 14.27.

### 2-Oxo-2H-chromen-7-yl 2-(3-chloro-2-methylphenyl)-1H-benzo[d]imidazole-5-carboxylate (5m)

Brown solid; yield: 74%; MP = 182–184 °C; IR (KBr, v_max_) 3326(NH), 3045 (C–H aromatic), 2990 (CH aliphatic), 1679 (C=O) cm^−1^;^1^H NMR (400 MHz, DMSO-*d*_6_) δ 10.11 (s, 1H, NH_Imidazole_), 8.33 (s, 1H, H_Ar_), 8.01 (d, *J* = 9.5 Hz, 1H, H_Ar_), 7.86 (d,* J* = 7.8 Hz, 1H, H_Ar_), 7.65 (d, *J* = 8.7 Hz, 1H, H_Ar_), 7.50 (d,* J* = 7.9 Hz, 1H, H_Ar_), 7.37 (d, *J* = 7.9 Hz, 1H, H_Ar_), 7.32 (d, *J* = 7.9 Hz, 1H, H_Ar_), 7.23 (d, *J* = 8.0 Hz, 1H, H_Ar_), 7.20 (s, 1H, H_Ar_), 7.04 (dd, *J* = 8.6, 2.4 Hz, 1H, H_Ar_), 6.32 (d, *J* = 9.5 Hz, 1H, H_Ar_), 2.26 (s, 3H, CH_3_). ^13^C NMR (101 MHz, DMSO-d_6_) δ 165.15, 161.61, 160.76, 155.80, 152.98, 147.74, 144.80, 142.21, 139.82, 137.47, 134.34, 130.86, 130.01, 127.46, 127.14, 126.92, 124.80, 122.29, 119.04, 115.78, 113.42, 113.15, 113.05, 102.00, 15.57.

### In vitro α-glucosidase inhibition assay

α-Glucosidase (*Saccharomyces cerevisiae*, EC3.2.1.20, 20 U/mg) and the substrate, p-nitrophenyl-β-d-glucopyranoside (p-NPG) were purchased from Sigma-Aldrich, and the assay was performed exactly according to our previous report^34,35^.

### Enzyme kinetic studies

The mode of inhibition of the most active compound **5k** identified with the lowest IC_50_ was investigated against α-glucosidase activity with different concentrations of *p*-nitrophenyl *α*-d-glucopyranoside (1–16 mM) as substrate in the absence and presence of sample **5k** at different concentrations (0, 2.7, 5.4, and 10.8 µM)^36,37^. A Lineweaver–Burk plot was generated to identify the type of inhibition and *K*_m_ value was determined from the plot between the reciprocal of the substrate concentration (1/[S]) and reciprocal of enzyme rate (1/V) over various inhibitor concentrations. Experimental *K*_i_ value was constructed by secondary plots of the inhibitor concentration [I] versus *K*_m_.

### Docking study

Docking study of the selected compounds **5c–e**, **5k**, and **5l–m** were performed exactly according to our previous reported work^33^ (Supplementary Information).

### Supplementary Information

Supplementary Information 1.

Supplementary Information 2.

## Data Availability

The datasets used or analyzed during the current study are available from the corresponding author on reasonable request.
