# Supplementary material for: Coumarin linked to 2-phenylbenzimidazole derivatives as potent α-glucosidase inhibitors
Source: Sci Rep. 2024 Mar 28;14:7408. doi: 10.1038/s41598-024-57673-z (PMC10978946; doi:10.1038/s41598-024-57673-z)
Supplement: Supplementary file 1 — Supplementary Information 1. [file 41598_2024_57673_MOESM1_ESM.docx]

**Support information**

**2-oxo-2H-chromen-7-yl 2-phenyl-1H-benzo[d]imidazole-5-carboxylate (5a)**

**
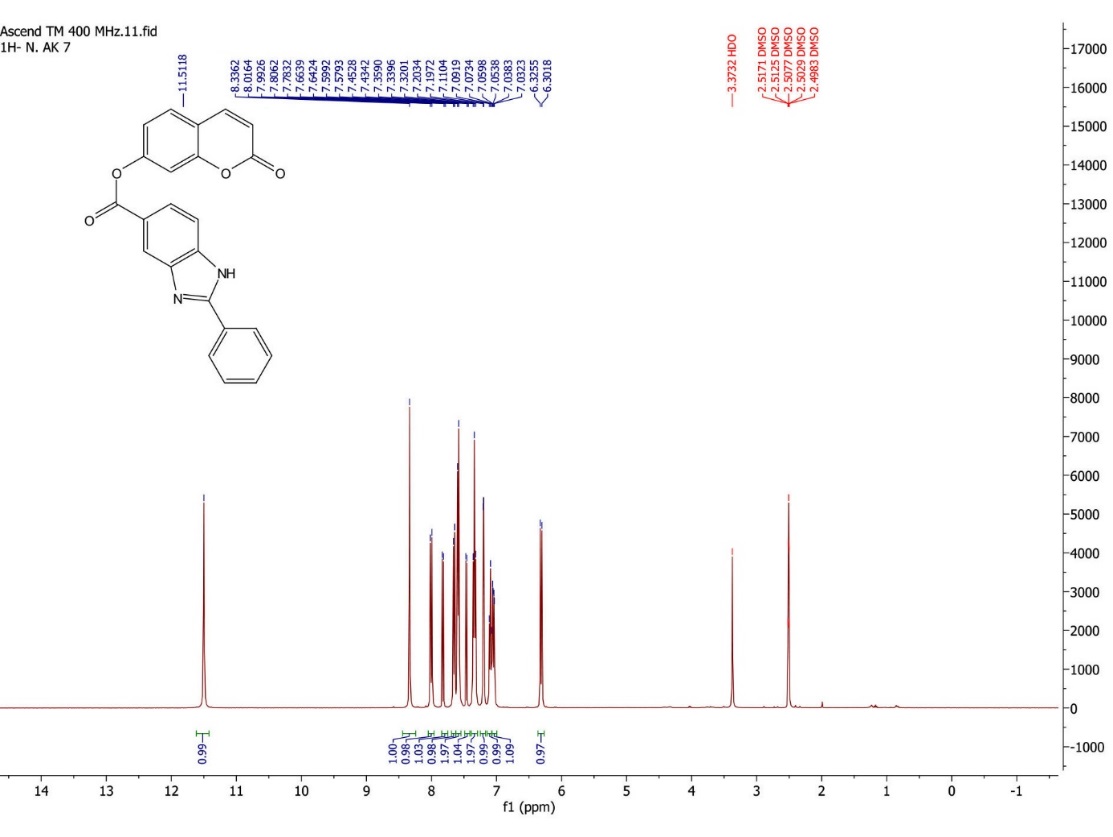
**

**
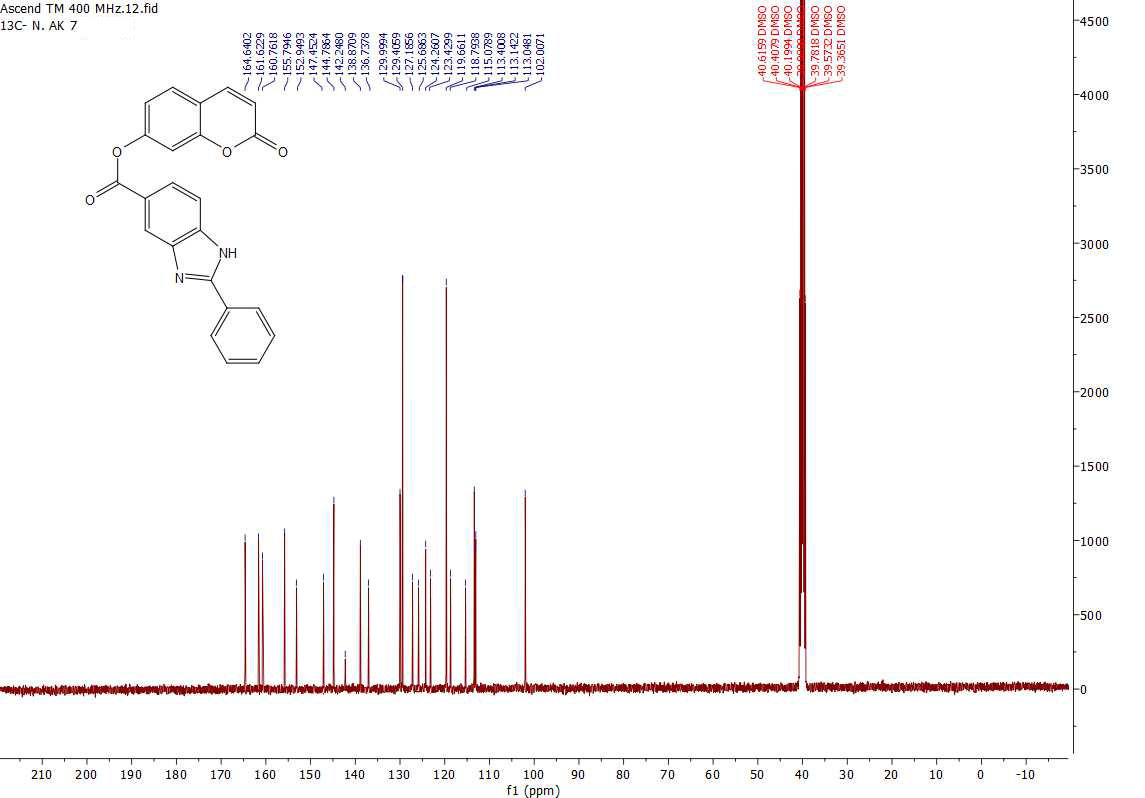
**

**2-oxo-2H-chromen-7-yl 2-(4-fluorophenyl)-1H-benzo[d]imidazole-5-carboxylate (5b)**

**
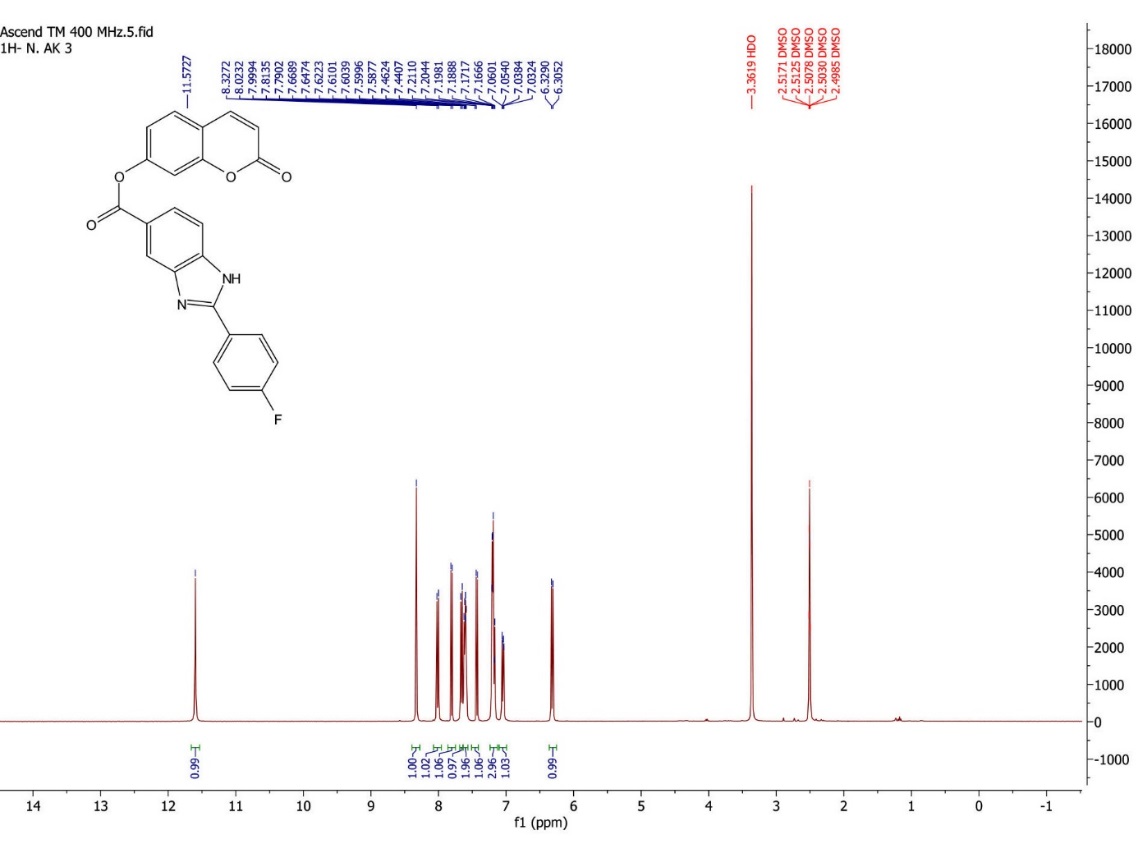
**

**
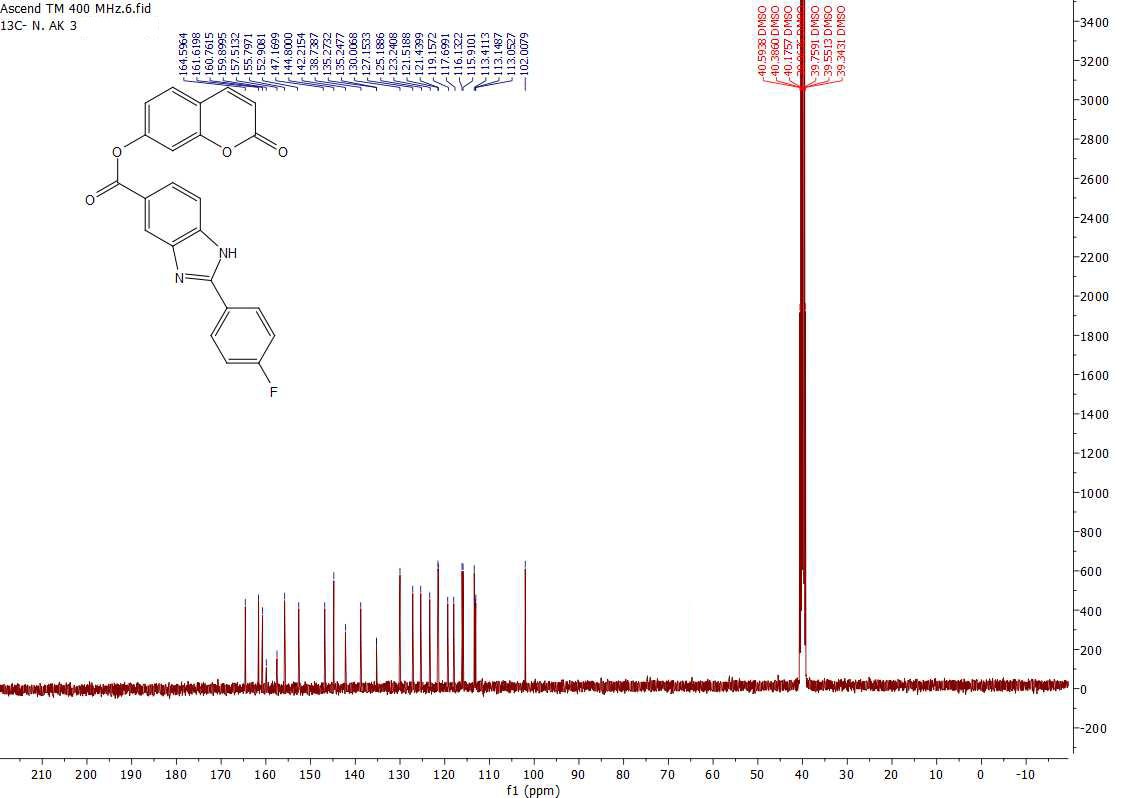
**

**2-oxo-2H-chromen-7-yl 2-(4-chlorophenyl)-1H-benzo[d]imidazole-5-carboxylate (5c)
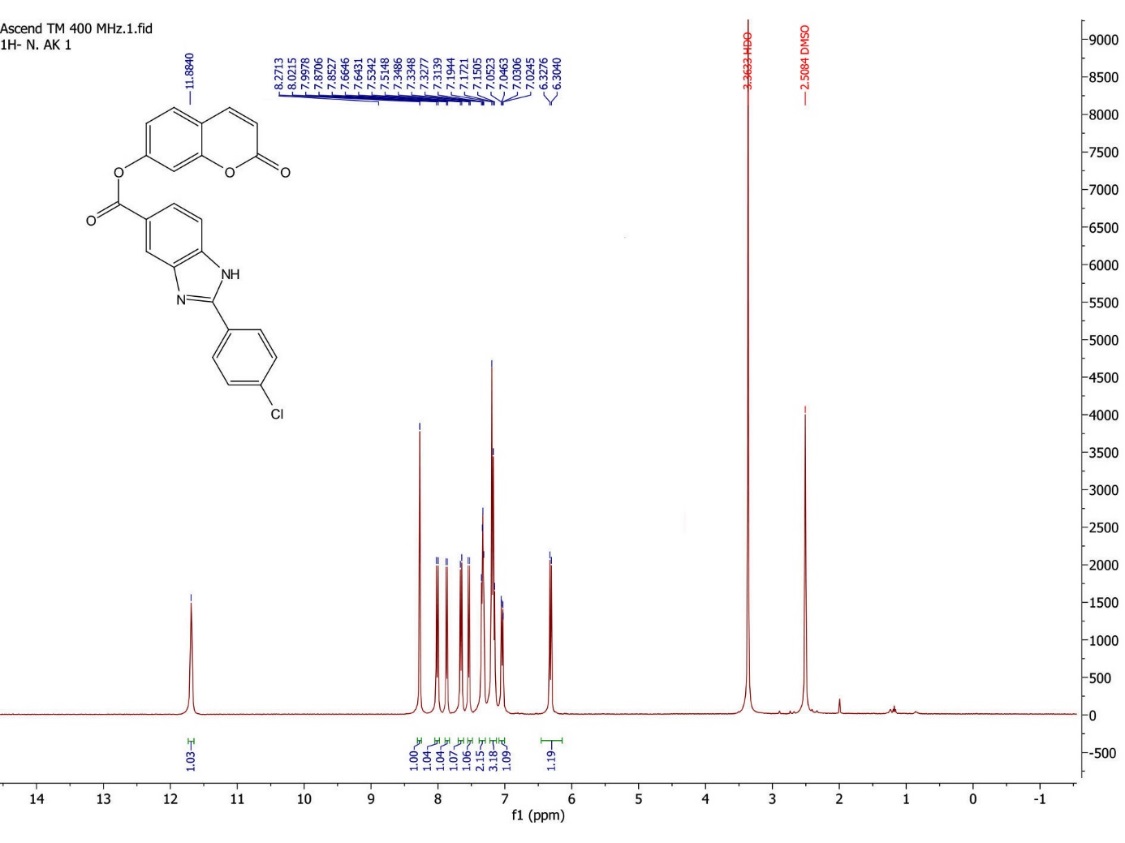
**

**
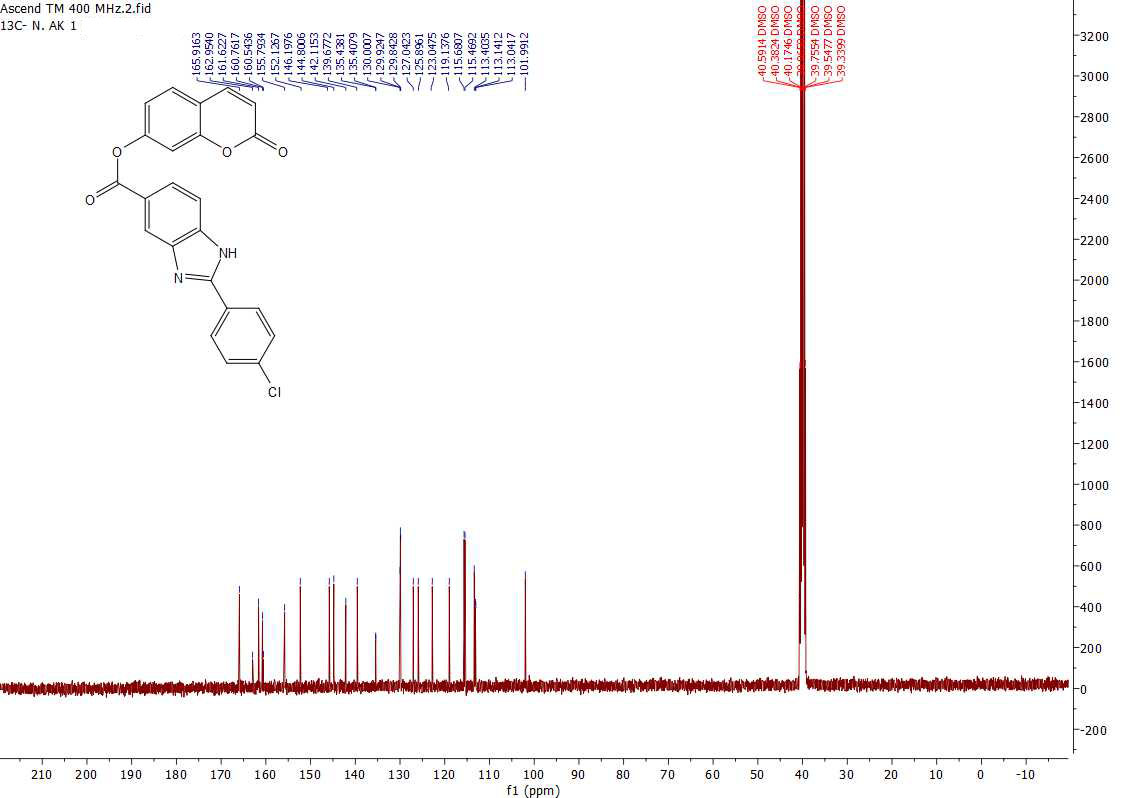
**

**2-oxo-2H-chromen-7-yl 2-(4-bromophenyl)-1H-benzo[d]imidazole-5-carboxylate (5d)**

**
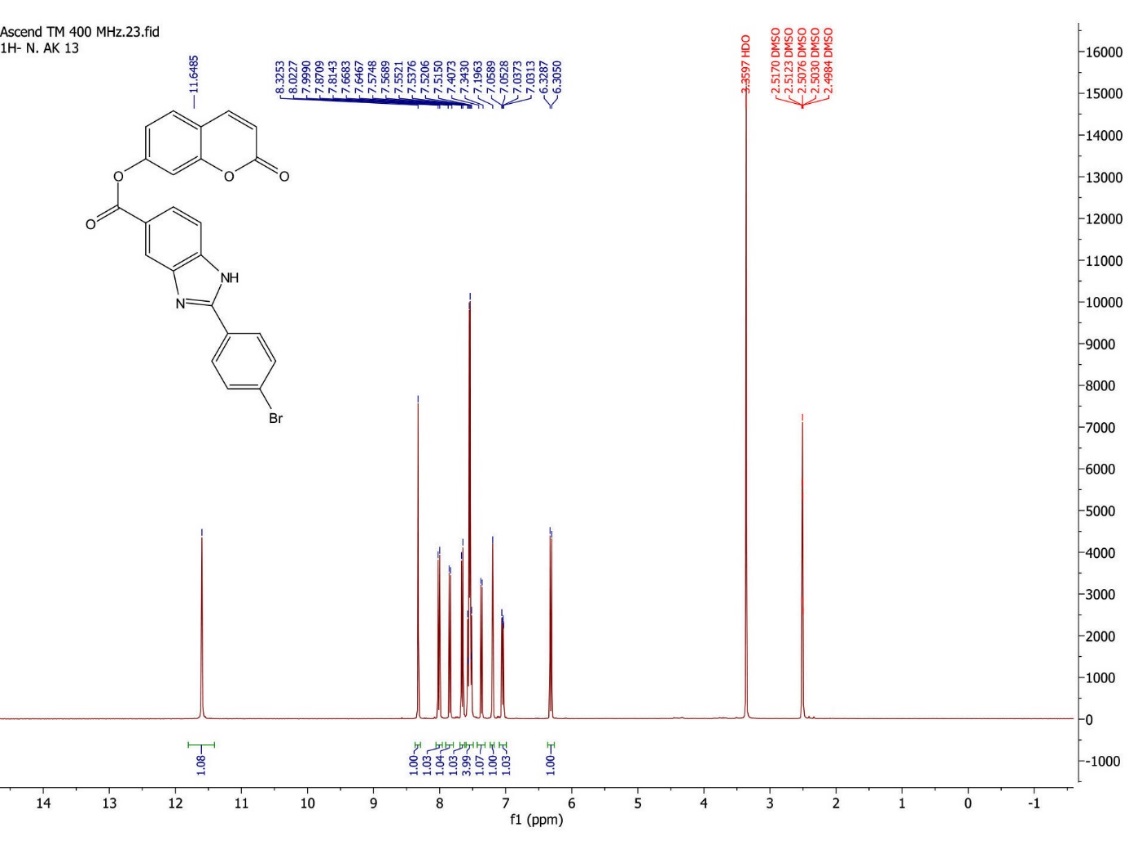
**

**
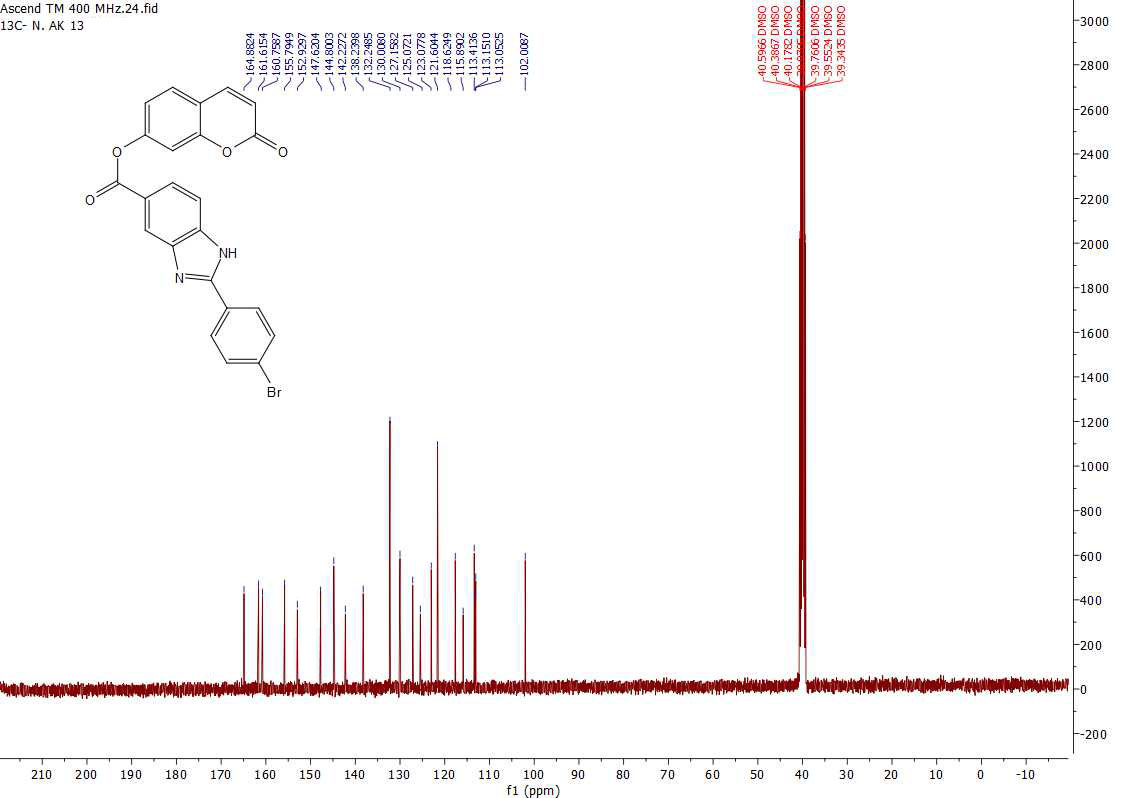
**

**2-oxo-2H-chromen-7-yl 2-(2-chlorophenyl)-1H-benzo[d]imidazole-5-carboxylate (5e)**

**
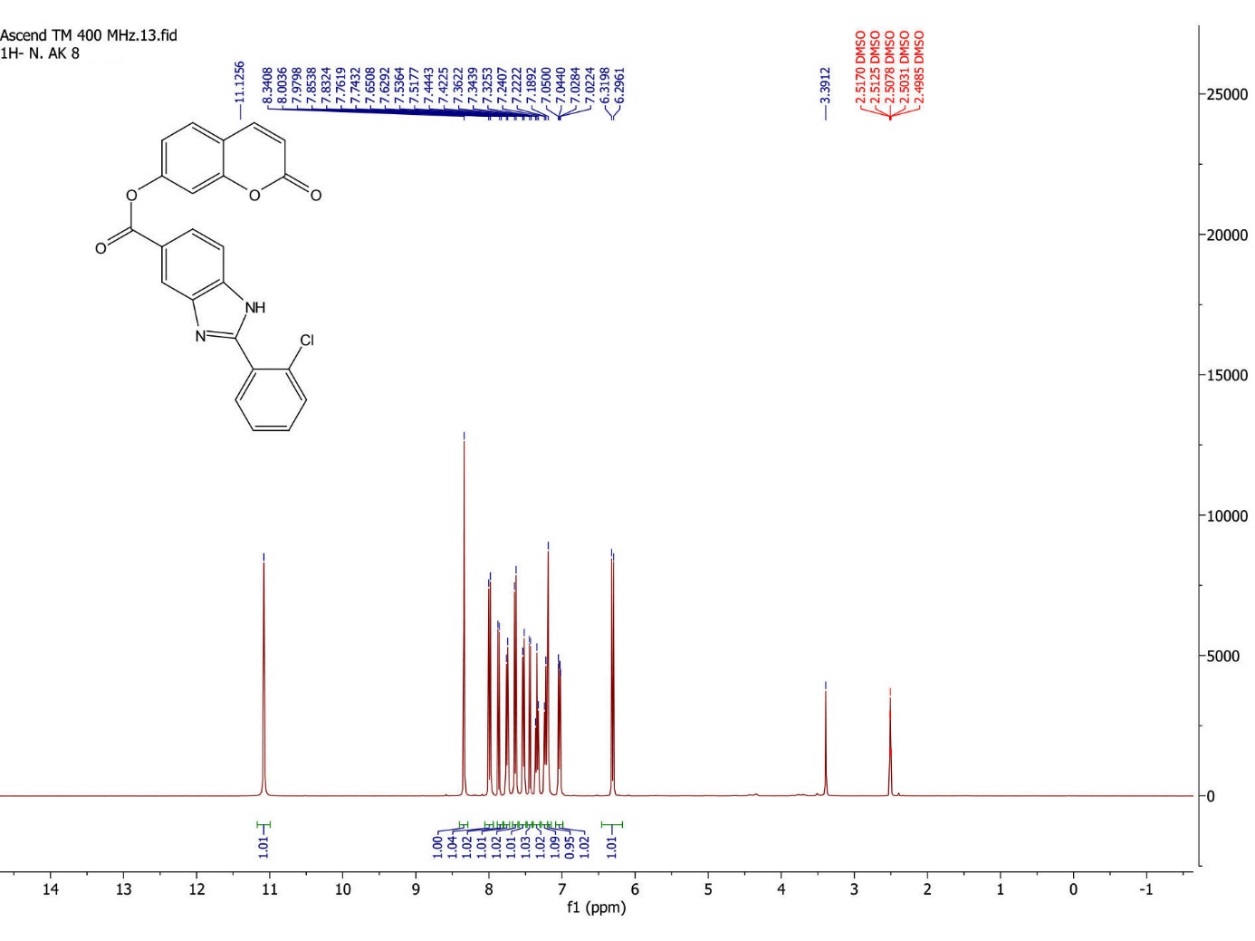
**

**
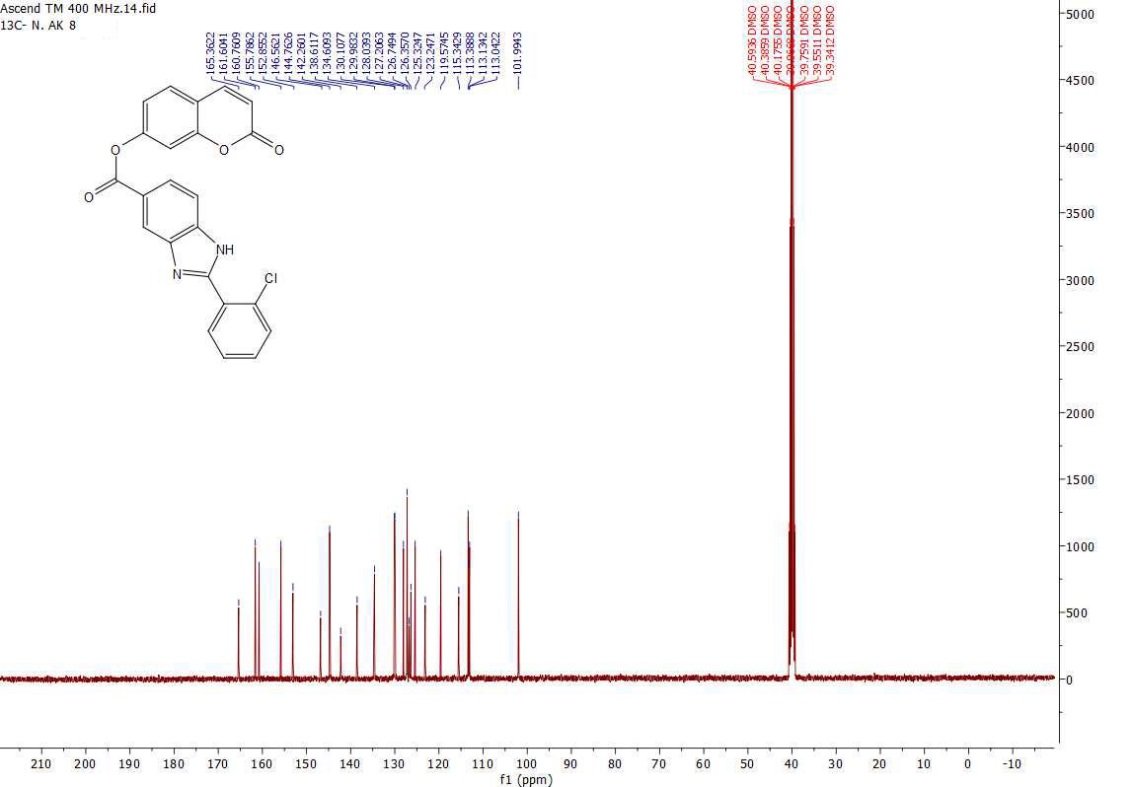
**

**2-oxo-2H-chromen-7-yl 2-(3-bromophenyl)-1H-benzo[d]imidazole-5-carboxylate (5f)**

**
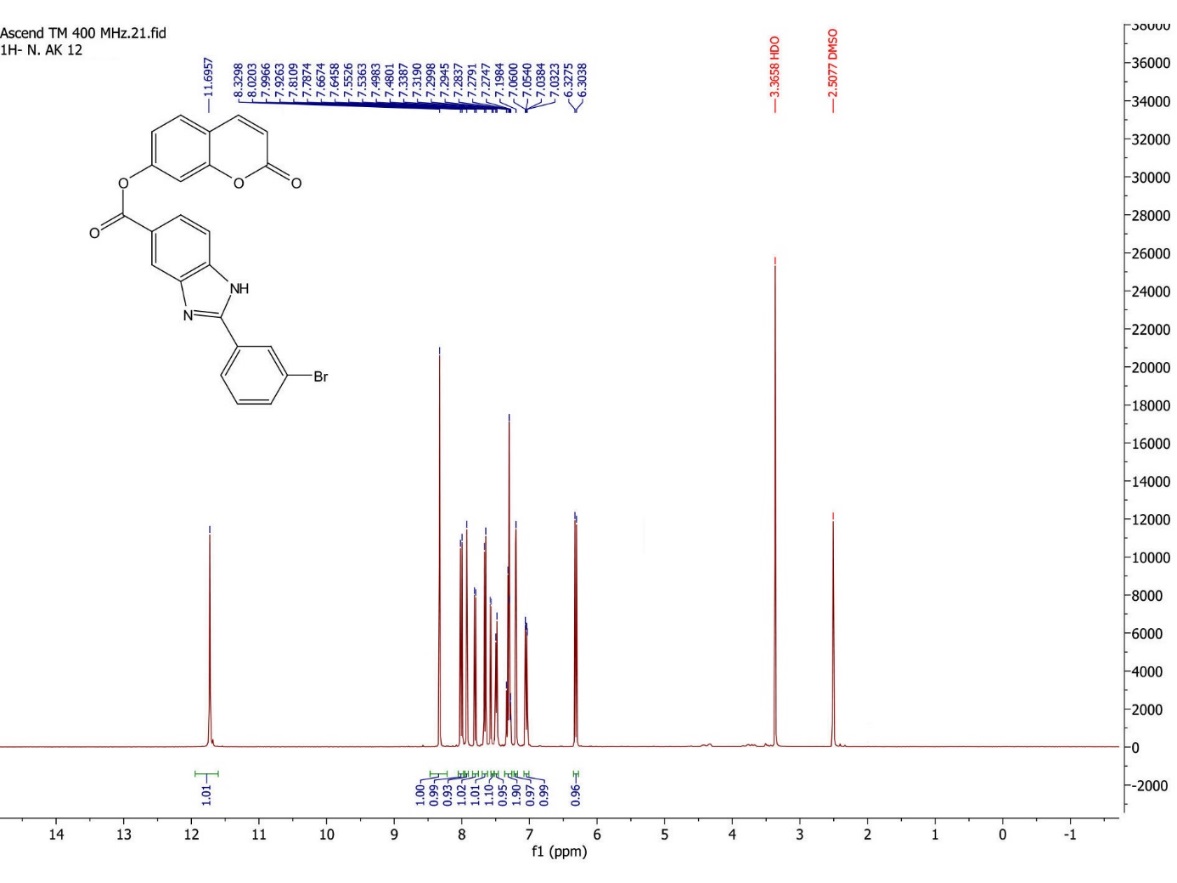
**

**
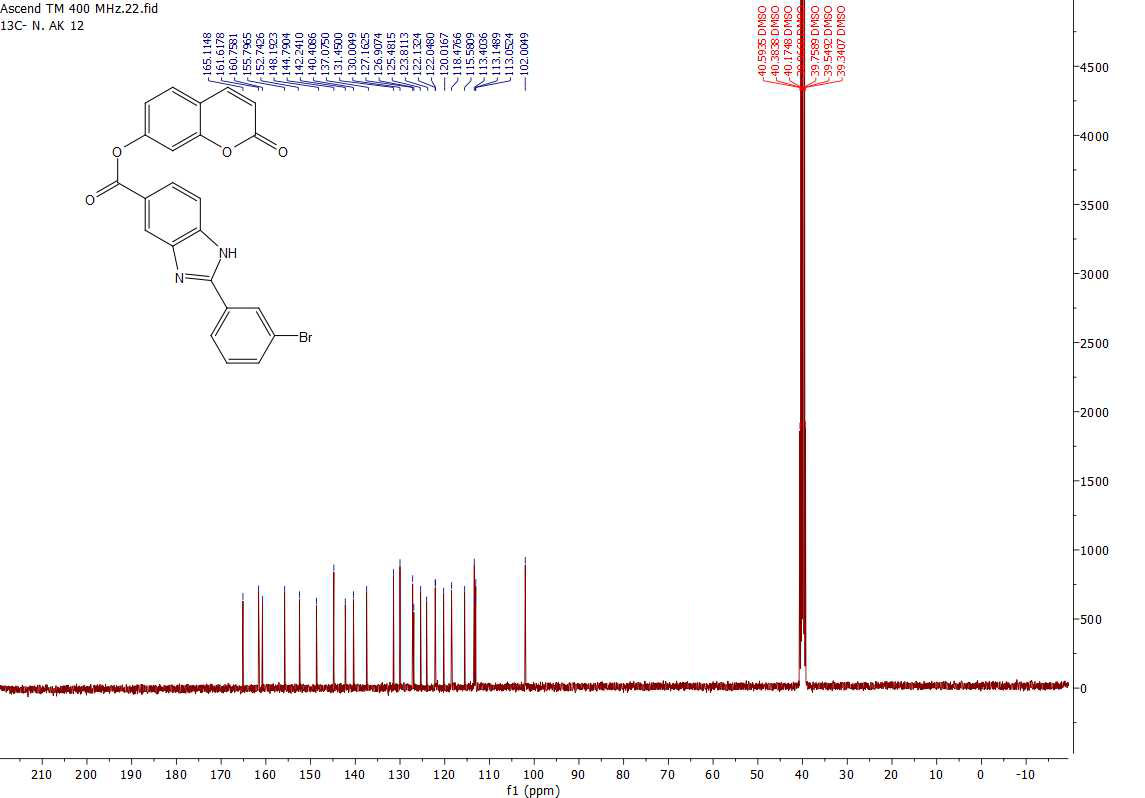
**

**2-oxo-2H-chromen-7-yl 2-(2,4-difluorophenyl)-1H-benzo[d]imidazole-5-carboxylate (5g)**

**
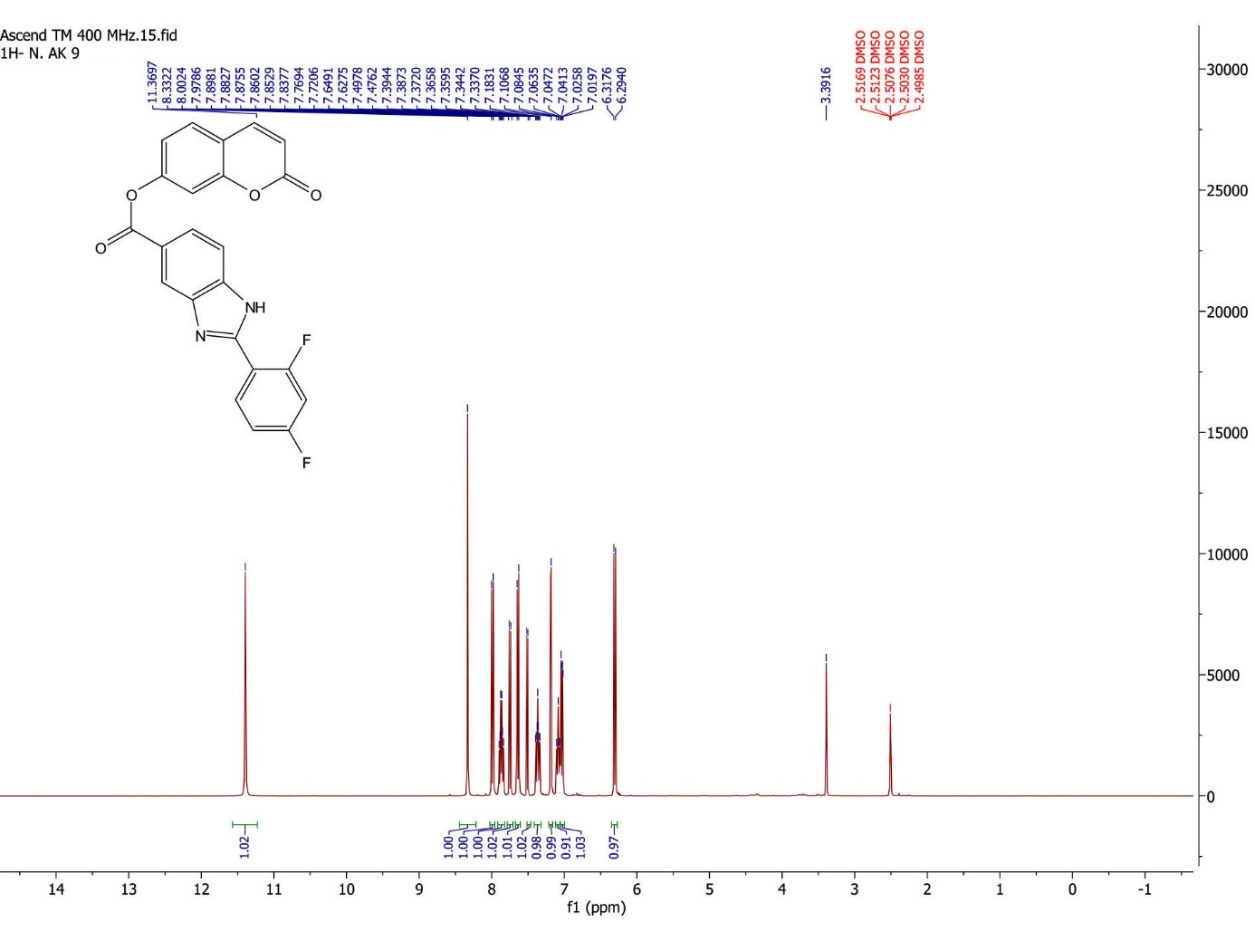
**

**
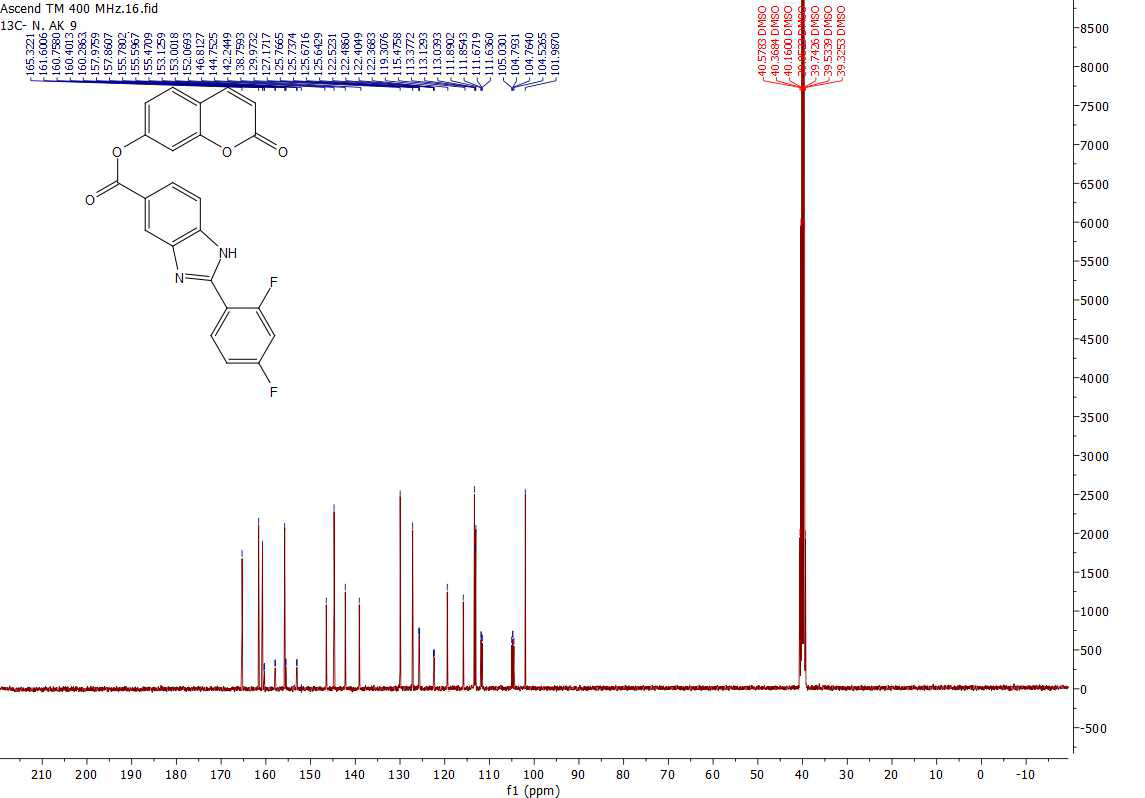
**

**2-oxo-2H-chromen-7-yl 2-(2,3-dichlorophenyl)-1H-benzo[d]imidazole-5-carboxylate (5h)**

**
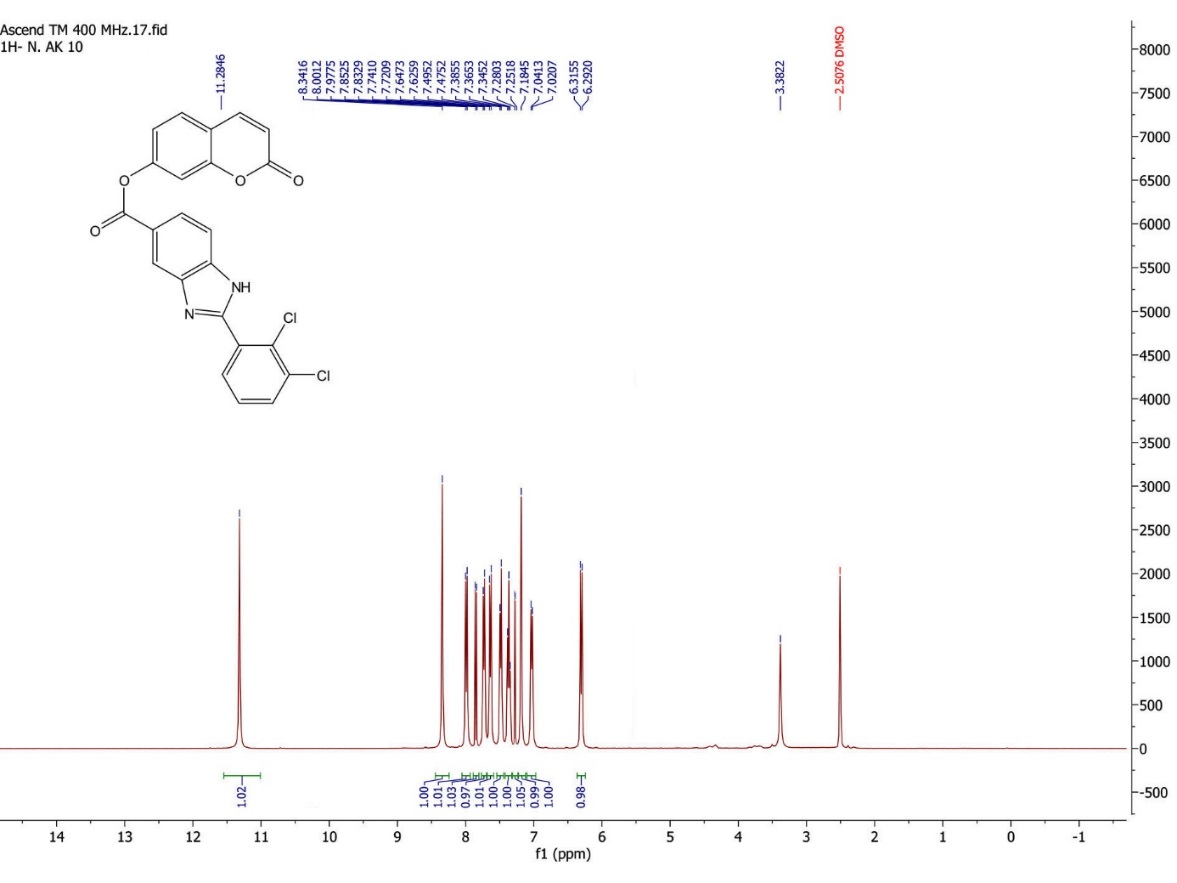
**

**
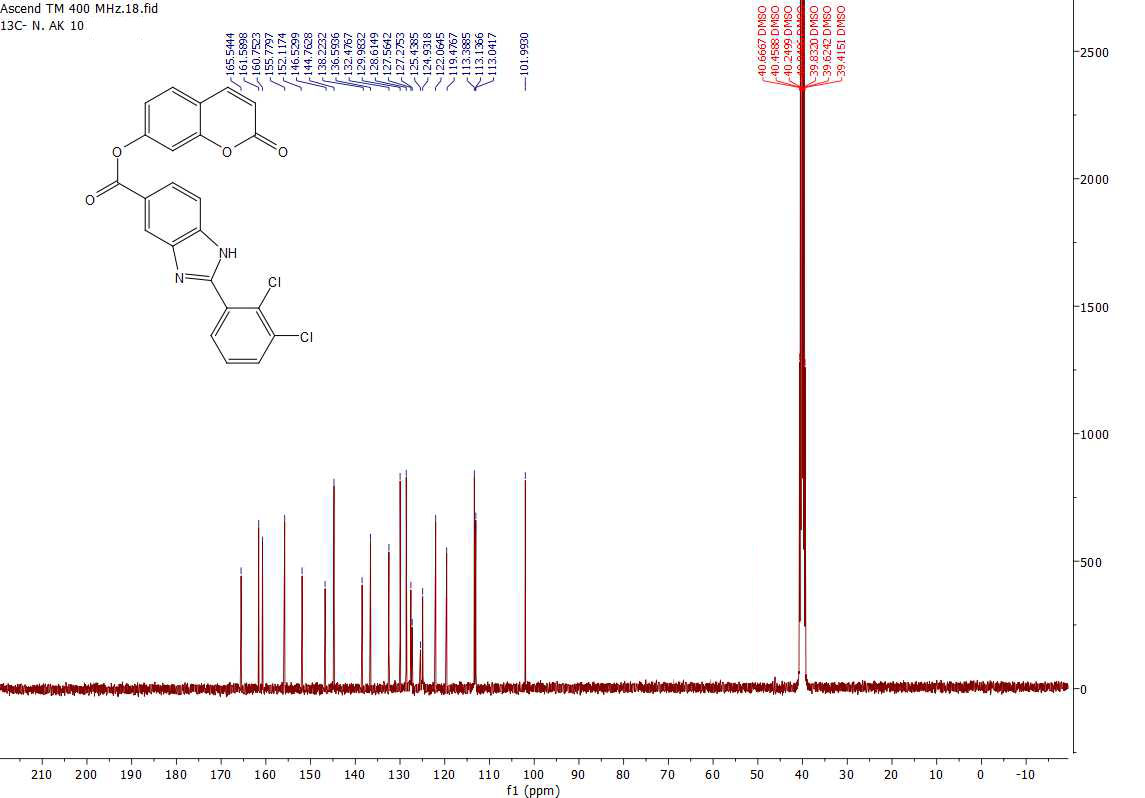
**

**2-oxo-2H-chromen-7-yl 2-(p-tolyl)-1H-benzo[d]imidazole-5-carboxylate (5i)**

**
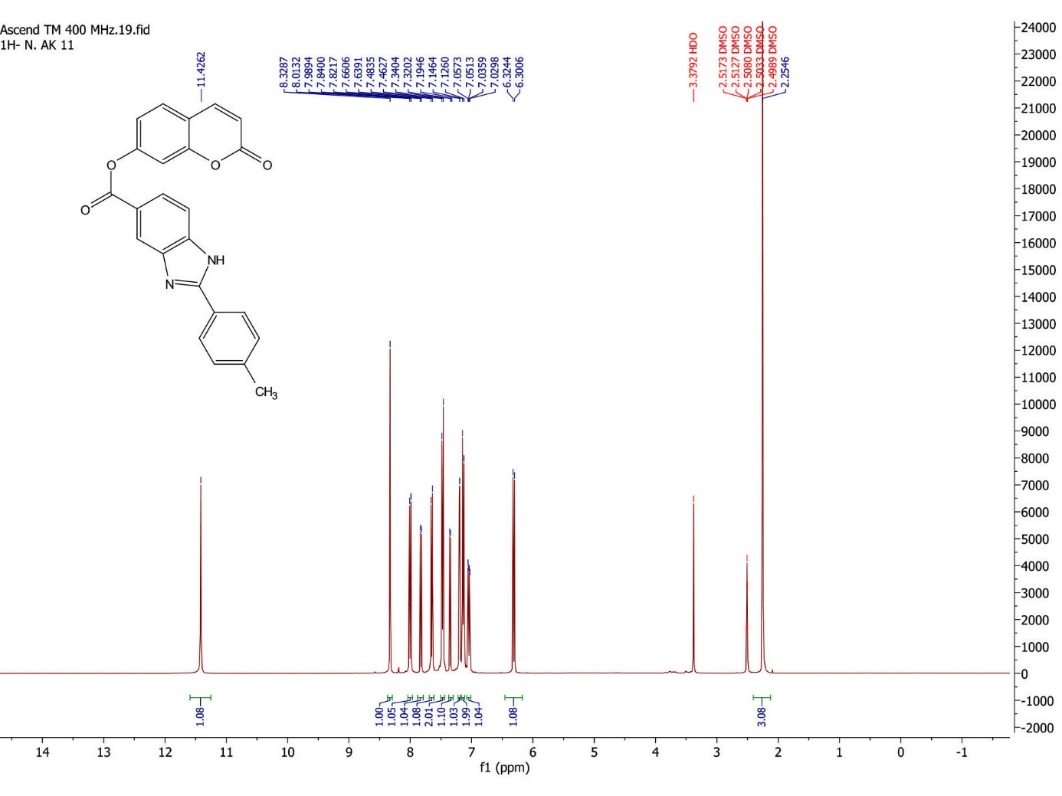
**

**
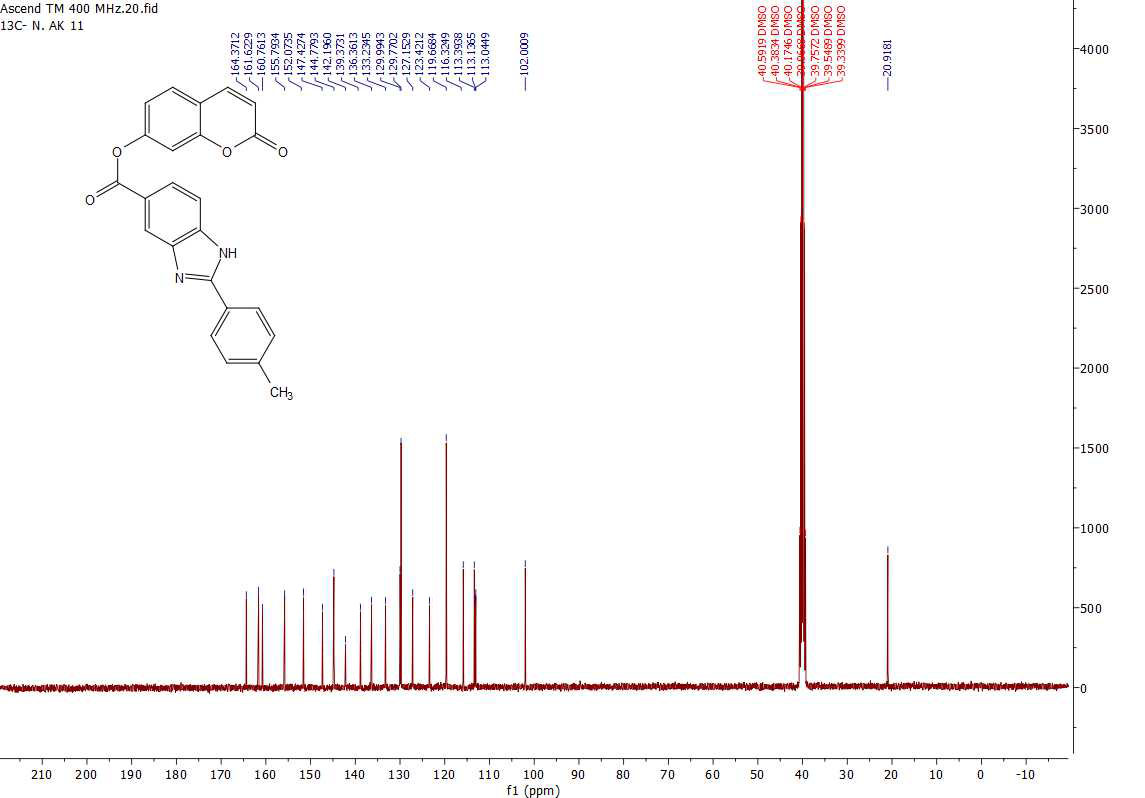
**

**2-oxo-2H-chromen-7-yl 2-(4-methoxyphenyl)-1H-benzo[d]imidazole-5-carboxylate (5j)**

**
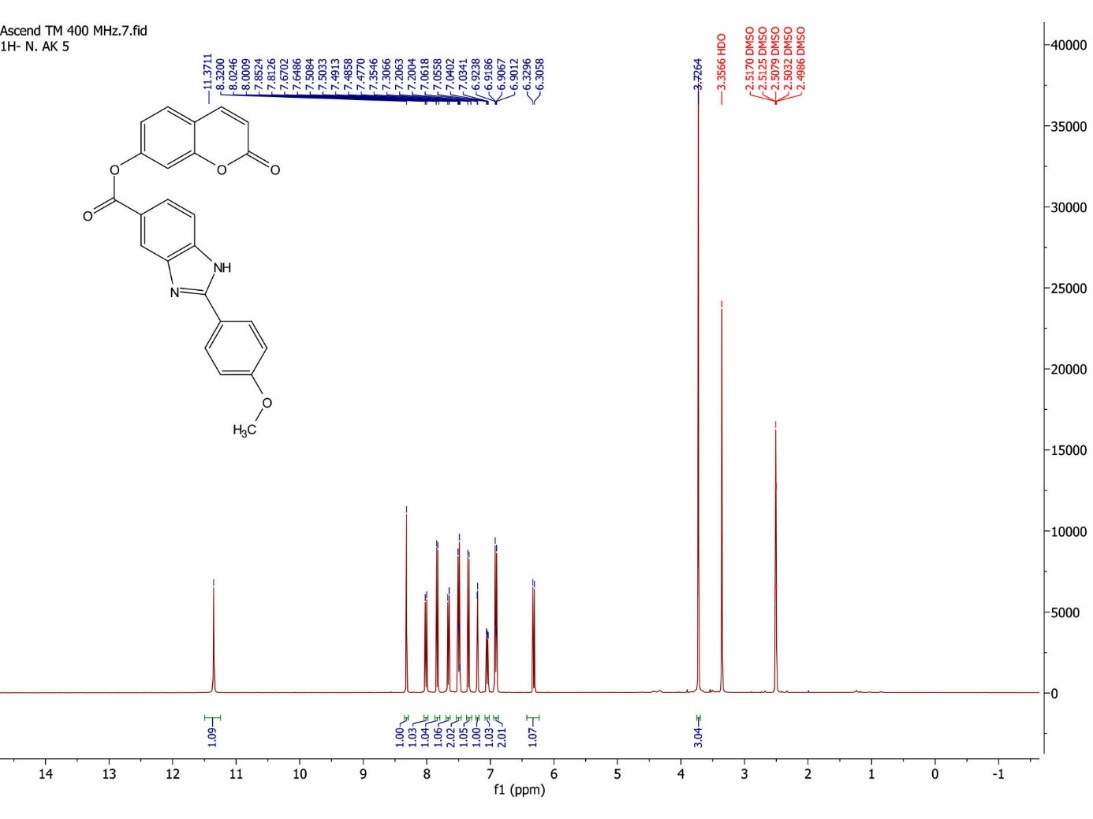
**

**
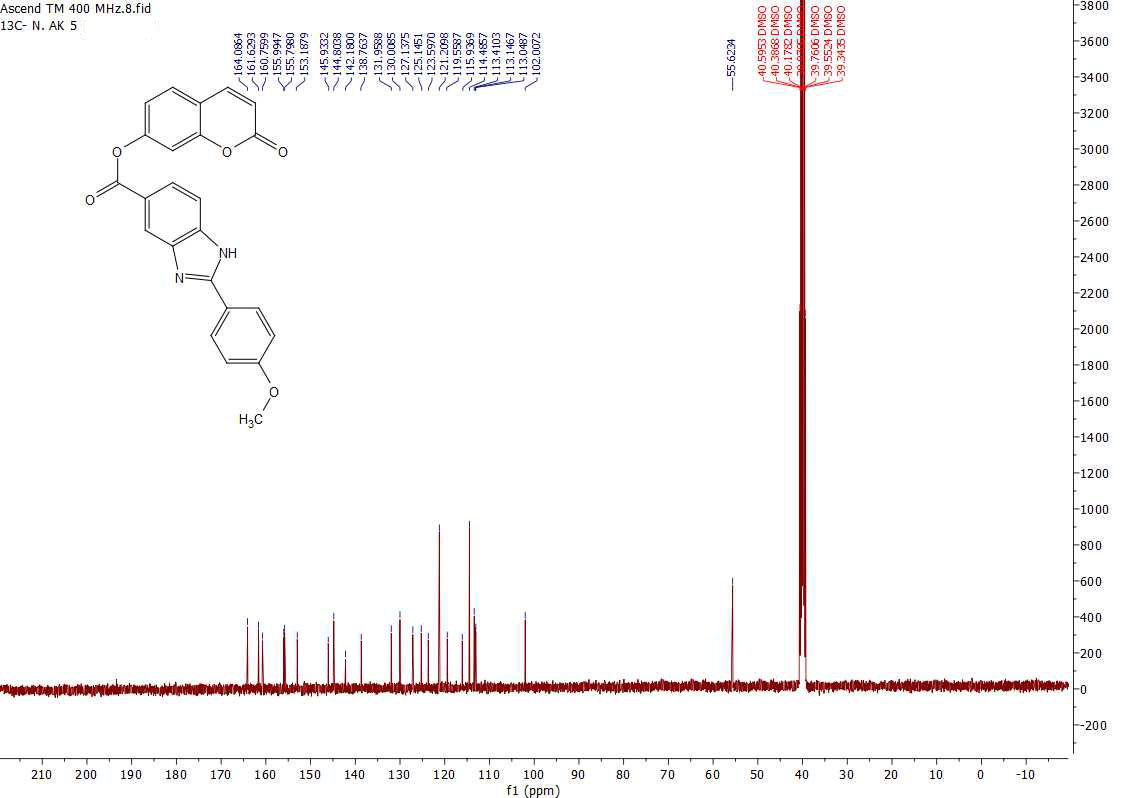
**

**2-oxo-2H-chromen-7-yl 2-(2-hydroxyphenyl)-1H-benzo[d]imidazole-5-carboxylate (5k)**

**
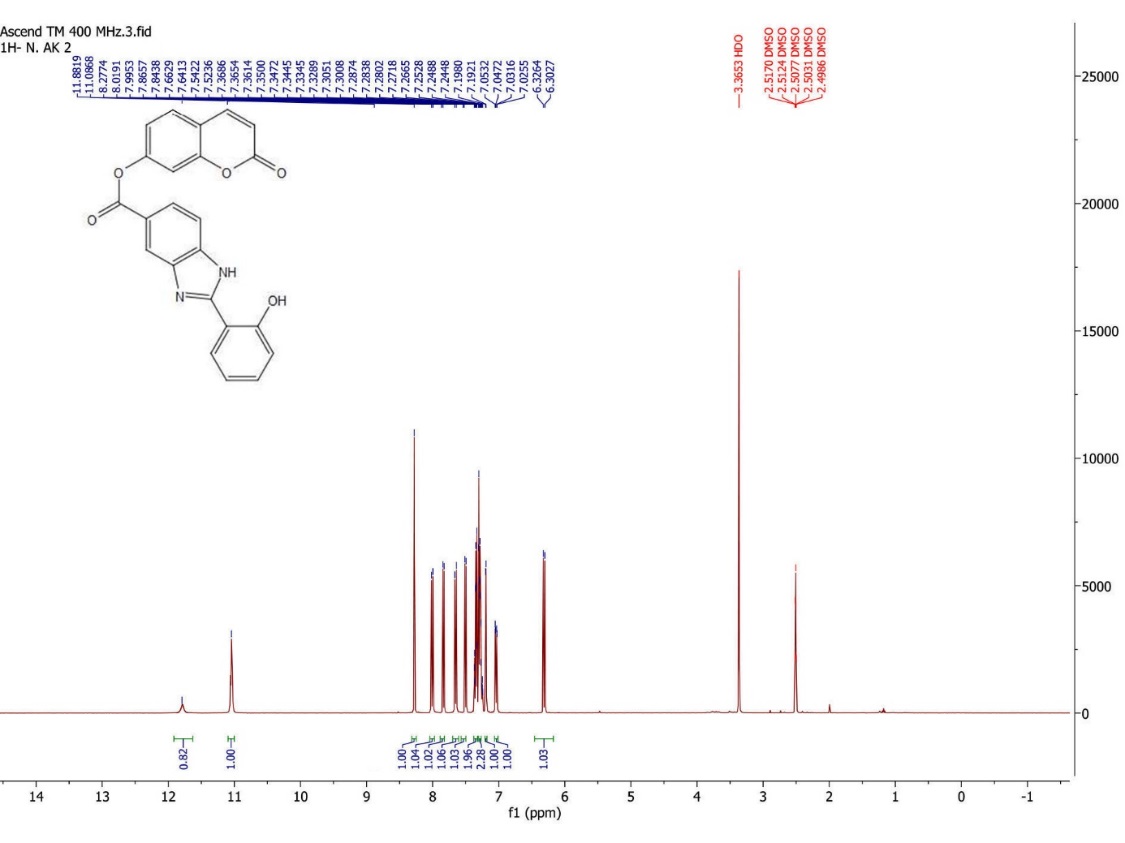
**

**
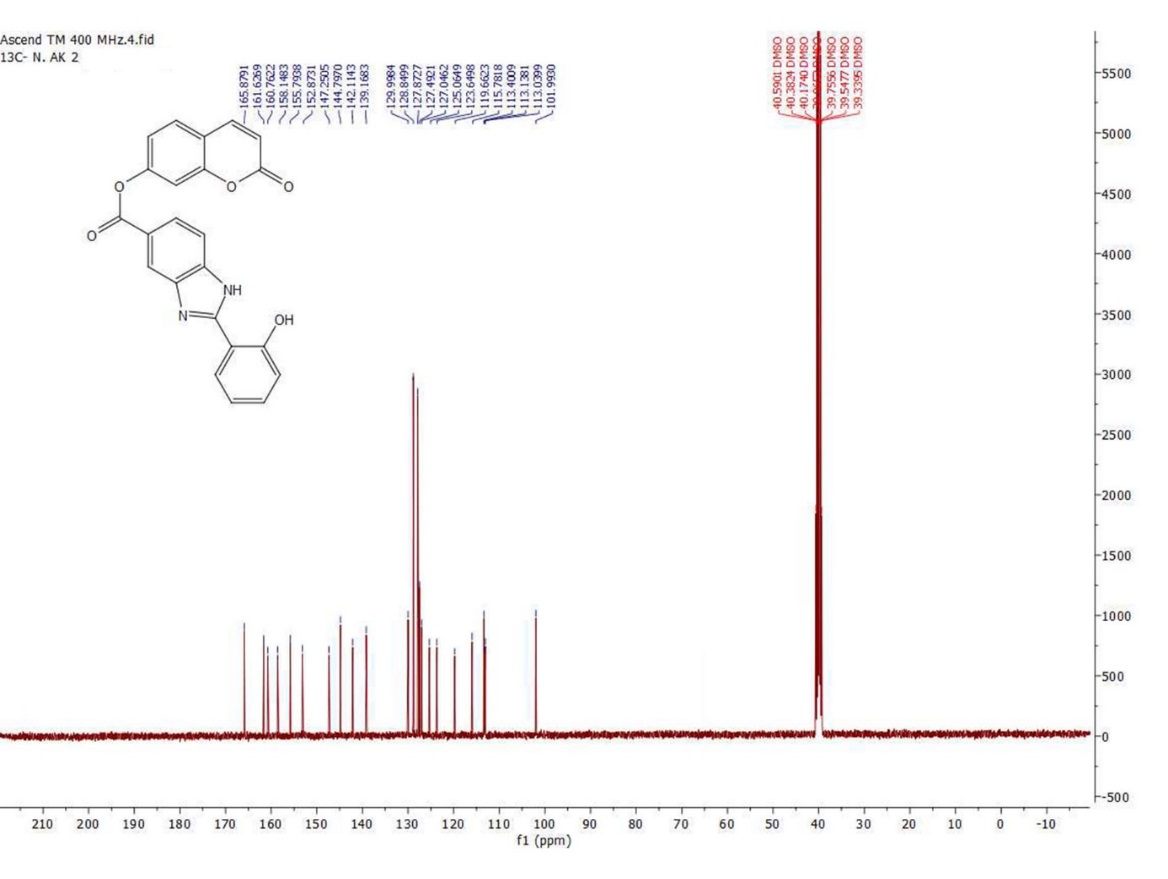
**

**2-oxo-2H-chromen-7-yl 2-(2-methyl-3-nitrophenyl)-1H-benzo[d]imidazole-5-carboxylate (5l)**

**
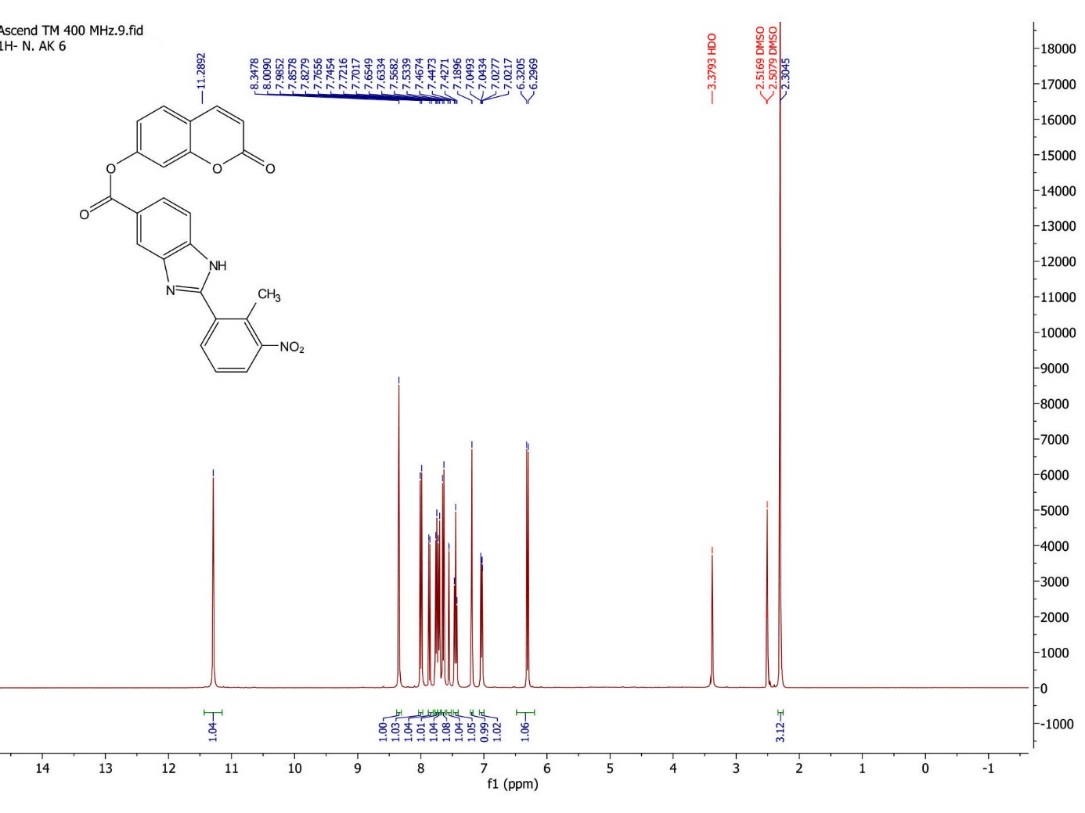
**

**
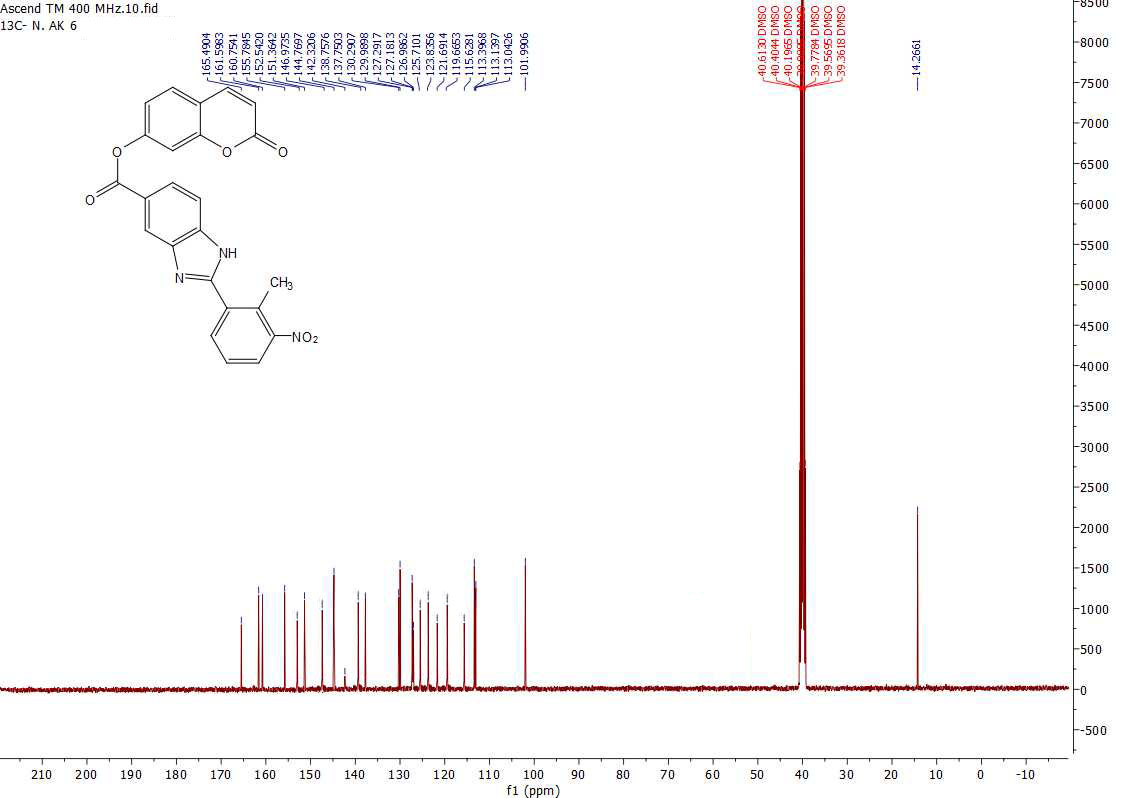
**

**2-oxo-2H-chromen-7-yl 2-(3-chloro-2-methylphenyl)-1H-benzo[d]imidazole-5-carboxylate (5m)**

**
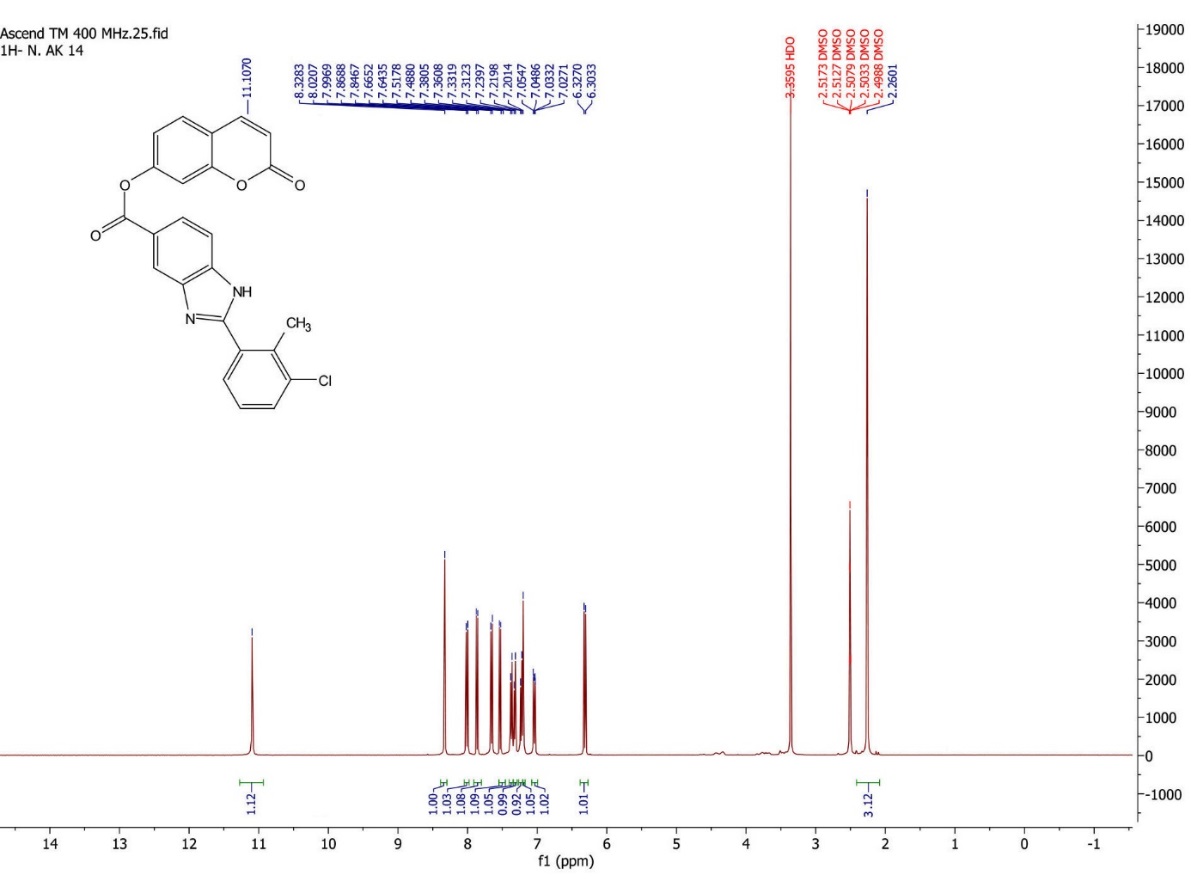
**

**
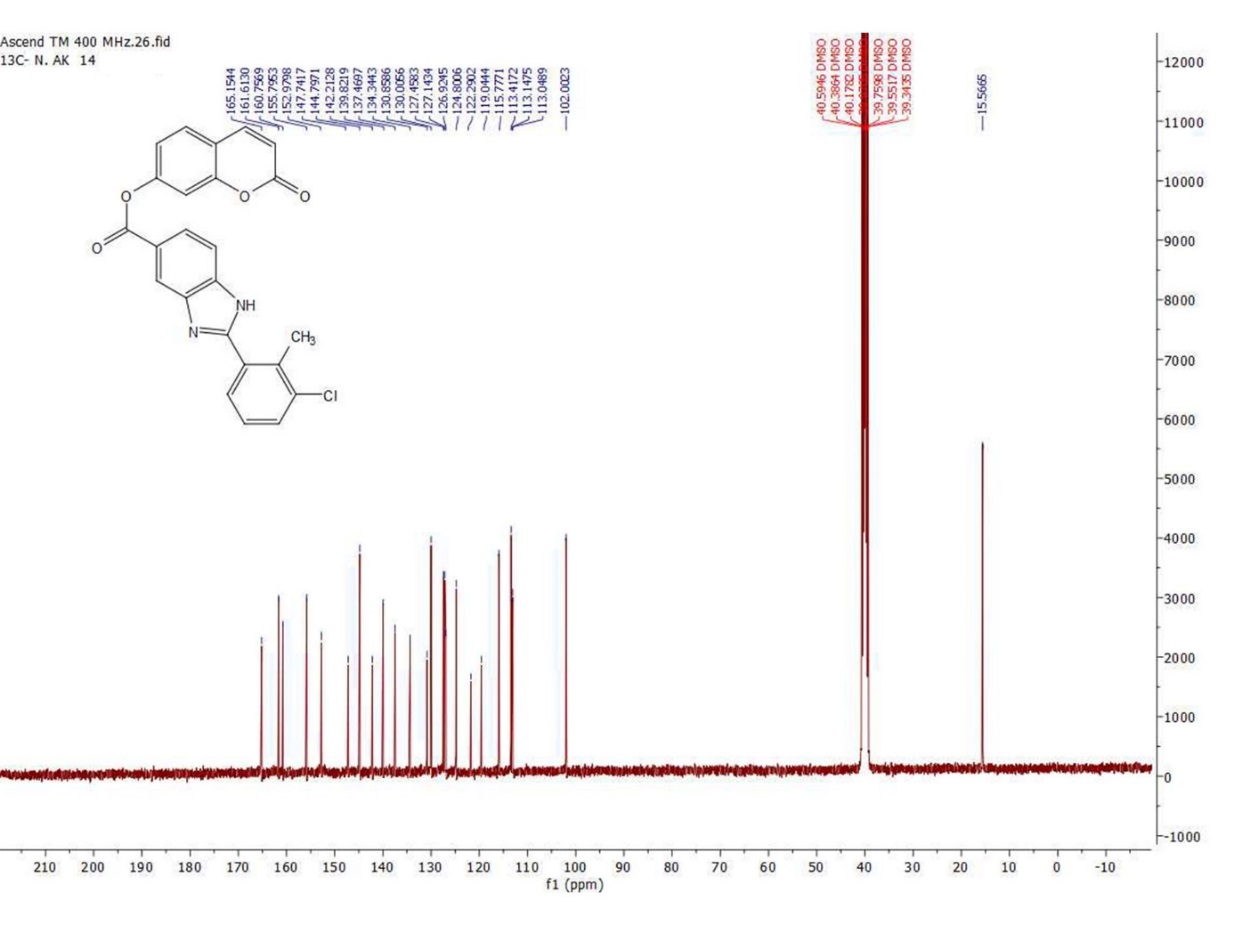
**
